# Too Sick to be True? Evaluating Potentially Problematic Diagnosis Coding Practices in Medicare's Patient‐Driven Payment Model

**DOI:** 10.1111/1475-6773.70084

**Published:** 2026-02-09

**Authors:** Harsha Amaravadi, Rachel A. Prusynski, Paul A. Fishman, Natalie E. Leland, Tracy M. Mroz

**Affiliations:** ^1^ Department of Health Systems and Population Health University of Washington Seattle Washington USA; ^2^ Department of Rehabilitation Medicine University of Washington Seattle Washington USA; ^3^ Department of Occupational Therapy University of Pittsburgh Pittsburgh Pennsylvania USA

**Keywords:** case mix adjustment, coding intensity, for‐profit, healthcare delivery, multimorbidity, reimbursement mechanisms, skilled nursing facilities

## Abstract

**Objective:**

To use a quasi‐experimental design to quantify changes in skilled nursing facility (SNF) diagnosis documentation associated with Medicare's Patient‐Driven Payment Model (PDPM). PDPM aims to promote patient‐centered care in skilled nursing facilities (SNFs) by matching reimbursement to patient characteristics, including clinical complexity, which is captured in part through documentation of diagnoses.

**Study Setting and Design:**

We used a difference‐in‐differences design to estimate PDPM's effects on SNF diagnosis documentation, including the number of diagnoses and clinical complexity scores via the Elixhauser comorbidity index. Hospital claims served as a non‐equivalent dependent variable control. Triple interaction terms in fixed effect linear models assessed variation by SNF profit status. Changes in the probability of recording five documentation‐sensitive conditions were estimated via marginal effects from generalized linear models.

**Data Sources and Analytic Sample:**

Secondary analysis of 100% Traditional Medicare claims (2018–2021), comprising over 4.8 million hospital‐to‐SNF episodes.

**Principal Findings:**

Compared against hospital claims from hospital‐SNF episodes, PDPM announcement was associated with 0.83 additional diagnoses on SNF claims, representing a relative increase of 7.1%. Similarly, Elixhauser scores increased by 0.88 points (relative 13.6%). We observed significant variation by profit status; when accounting for anticipatory behavior, profit status was associated with an additional relative 2.8% in diagnoses and 4% in Elixhauser points. PDPM was also associated with increased probability of documenting all five documentation‐sensitive conditions: 3.9 percentage points (pp) for chronic pulmonary disease, 5.0 pp for complicated diabetes, 2.8 pp for heart failure, 7.3 pp for obesity, and 9.8 pp for weight loss (all reported *p* < 0.001).

**Conclusions:**

PDPM was associated with increased coding intensity across multiple measures—and more so in for‐profit SNFs—highlighting the need to further evaluate whether SNFs are accurately documenting or falsely inflating clinical complexity. Sustaining Medicare's payment accuracy will require continued monitoring of diagnosis coding behavior and its alignment with actual *clinical* complexity.

## Callout Boxes

- What is known about this topic
  ○ Historically, certain SNFs (e.g., profit‐motivated SNFs) have changed behaviors in response to Medicare payment policy, but precise estimation of such behavioral changes remains limited.
  ○ The Patient‐Driven Payment Model (PDPM) fundamentally changed the way Skilled Nursing Facilities (SNF) were paid under Medicare to promote patient‐centricity while remaining budget‐neutral.
  ○ PDPM aims to reimburse SNFs appropriately for the work required to care for clinically complex patients, where reimbursement rates are determined partly based on diagnoses rather than therapy volume provided.
- What this study adds
  ○ For all Traditional Medicare hospital‐SNF episodes between 2018–2021, coding intensity increased more than was expected after PDPM announcement (relative 7.1% and 13.6% increase in diagnosis count and Elixhauser score, respectively).
  ○ For‐profit SNFs documented diagnoses significantly more than not‐for‐profits, and among all SNFs, the probability of recording five documentation‐sensitive conditions increased significantly after PDPM announcement.
  ○ Changes in documentation may reflect both false inflation of complexity (upcoding) and new efforts to code existing patient complexity.

## 1 Introduction

SNFs provide post‐hospital transitional rehabilitation and nursing care to approximately 1.2 million Medicare beneficiaries annually. From Medicare's perspective, skilled nursing facilities (SNFs) are a highly scrutinized setting due to their substantial cost, approximately $25 billion in 2023, and their central role in caring for older adults, who represent the vast majority of SNF users [1, 2, 3]. On October 1, 2018—the start of Fiscal Year 2019—Medicare finalized the Patient‐Driven Payment Model (PDPM) through rulemaking, although the policy was not implemented for SNFs until 1 year later, on October 1, 2019. PDPM replaced the Resource Utilization Groups Version IV (RUG‐IV) reimbursement system. The RUG‐IV system calculated SNF payments based primarily on rehabilitation therapy volume (i.e., therapy minutes per week) and was criticized for leading to unnecessary or excessive rehabilitation therapy provision and being agnostic of patient clinical needs [4, 5, 6]. Under the RUG‐IV system, SNFs were not adequately reimbursed for caring for clinically complex patients who could not tolerate high‐volume rehabilitation. Accordingly, PDPM was designed to move away from volume‐based payments by removing incentives for therapy and aligning reimbursement with patient complexity and care needs [4]. Some research has identified reductions in therapy following PDPM, but research robustly quantifying how PDPM has affected coding intensity (i.e., the tendency of facilities to increase administrative documentation of patient complexity) remains limited. A prior cross‐sectional study found variable changes in documentation of some diagnoses after PDPM [7]; however, this study did not have an adequate comparison group to establish a counterfactual for estimating the extent of documentation changes after PDPM. Our study adds to the literature by quantifying changes in coding intensity using a longitudinal approach that accounts for underlying trends in case mix over time with a control arm.

Under PDPM, reimbursement rates are calculated based on patients' clinical characteristics (i.e., diagnoses, functional ability, and receipt of certain services) rather than volume of therapy services [8]. The clinical information used in PDPM reimbursement is recorded on mandated clinical assessments and the SNF claims billed to Medicare. SNFs may use information received from the hospital, including patient diagnoses, to complete documentation. PDPM differs from the prior RUG‐IV model in that clinical characteristics are incorporated into every component of care that influences reimbursement [5, 8]. The five components of care that are summed to calculate PDPM reimbursement rates are: nursing, physical therapy, occupational therapy, speech language pathology, and non‐therapy ancillary (NTA). For the first four components, diagnosis codes map to clinical categories that dictate payment adjustments that substantially influence overall reimbursement rates. Conversely, the NTA component is calculated based on the presence of specific comorbidities and services related to those comorbidities (e.g., wound care, parenteral feeding, and IV usage) that each garner different numbers of points based on their anticipated costs of care [8, 9, 10]. Ultimately, PDPM is a highly specified algorithm based on patient clinical information, which attempts to differentiate patients based on complexity [8, 9, 11].

The intent of PDPM is to pay SNFs appropriately for the care required to treat complex medical issues. However, in practice, payments are operationalized based on how well a SNF documents clinical complexity on claims and assessments, regardless of whether or not they deliver appropriate care. This paradox is an inherent limitation of reimbursement based on administrative data and has been brought up specifically for PDPM, underscoring the need to quantify the potential mismatch between documentation in administrative data and clinical reality [7, 12, 13, 14]. Changes in coding intensity represent two scenarios: (i) new emphasis on accurate coding of an existing complex population that had not been well‐documented previously [15, 16] or (ii) a false inflation of complexity (i.e., “upcoding”), whereby facilities submit an exaggerated picture of patient complexity to capitalize on financial incentives [13, 17]. The former represents the spirit of the policy, for example, rewarding a SNF which has been treating an existing complex patient pool but did not have the resources or incentive to document the complexity thoroughly, while the latter may represent potentially problematic upcoding.

The issue of upcoding in Medicare reimbursement, and for SNFs specifically, is not new and requires continuous monitoring efforts to ensure public programs are not being misused [13, 17, 18, 19, 20]. The standards of care in SNFs are not as clearly delineated as in other post‐acute settings—that is, SNF providers have a fair amount of discretion in determining appropriate provision of services [21]. Additionally, SNFs have historically made operational changes in response to payment policies [22]. For example, Bowblis et al. studied how SNFs changed care provision behavior in response to financial incentives introduced in the RUG‐IV system, implemented in 2011 [21]. They found that minutes of therapy provided increased in response to the volume‐based financial incentives of RUG‐IV, and there was no evidence of increased patient complexity [18]. We have already seen evidence of reduced therapy volumes in response to changes in incentives for therapy provision under PDPM [7, 23]. Accordingly, we hypothesize that PDPM will be associated with increased coding intensity as well.

Furthermore, our work adds to the literature on variation in coding intensity based on facility characteristics like profit status. To explore the potential for upcoding, we also examine documentation changes after the PDPM announcement for five documentation‐sensitive conditions and examine differences in changes in complexity documentation by SNF profit status. We hypothesize that (i) PDPM led to increased coding intensity, particularly for common chronic conditions, and that (ii) for‐profit SNFs are more likely to increase documentation as compared to not‐for‐profit SNFs.

Our study will support ongoing refinement of SNF payment policy under Medicare, as well as broader policies that tie reimbursement to patient complexity, such as risk adjustment within SNF Value‐Based Purchasing. Existing research unrelated to PDPM has already highlighted concerns around the potential inflation of diagnoses codes to enhance risk adjustment among Medicare Advantage SNFs [20]. The concept of coding intensity originates from the Medicare Advantage literature, and PDPM may have introduced similar incentives into SNF reimbursement for Traditional Medicare patients. Additionally, our work may highlight the need for targeted payment strategies across SNFs of varying profit status, something that has been called for in progressive payment policy design [22, 24, 25].

## 2 Study Data and Methods

We conducted a secondary analysis of 100% of Traditional Medicare administrative data from 2018 through 2021. The University of Washington institutional review board approved this study with a waiver of informed consent.

We conducted a difference‐in‐differences analysis with a non‐equivalent dependent variable (NEDV) control, comparing clinical complexity outcomes between SNF (treatment) and hospital (control) claims before and after PDPM announcement. To create our unit of analysis, beneficiary‐level hospital‐SNF care episodes, we matched individual beneficiaries' hospital claims to SNF claims that occurred within 3 days of hospital discharge. Given that SNFs use hospital discharge records as the primary source for diagnosis documentation [26], and the transition from hospital to SNF is typically short (1–2 days), hospital claims are an appropriate control to estimate the changes in coding intensity tied to PDPM.

### 2.1 Data Sources

The primary data source for this study was the Medicare Provider Analysis and Review (MedPAR) Research Identifiable Files, focusing on index hospitalizations that resulted in a SNF admission within the 50 states and the District of Columbia. We linked these records to the Medicare Master Beneficiary Summary File for coverage verification and patient demographics. Facility and market characteristics used for model adjustment were obtained from public data sources, including the CMS Provider of Services File, Nursing Home Compare (Provider Data Catalog), Long‐Term Care Focus, the Area Health Resource File, and USAFacts.

### 2.2 Study Population and Time Period

We analyzed inpatient hospital claims from 2018 to 2021 for all Traditional Medicare beneficiaries, ages 18+, who had continuous enrollment in Medicare Part A for 3 months following the index hospitalization, who did not leave the hospital against medical advice, and who transitioned from a hospital to a SNF within 3 days. Due to the three‐month continuous Medicare Part A enrollment requirement, the latest verifiable hospital discharge occurred on September 30, 2021. We exclude observations from SNFs located within hospitals, which are likely to share coding and billing guidance with the hospital, but which represent only 3% of the sample.

### 2.3 Study Design

We used a difference‐in‐differences design with a non‐equivalent dependent variable (NEDV) control to examine changes in coding intensity between hospital and SNF claims before and after PDPM announcement. An NEDV control is a useful analytic strategy for quasi‐experimental designs when a traditional control group is not available and provides additional analytic benefits for reducing confounding [27, 28]. As PDPM was finalized and then later implemented uniformly at distinct timepoints, there are no unexposed patients. An NEDV is an outcome that is separate but related to the primary study outcome and is also derived from the exposed group. An NEDV should be affected by the same time‐varying confounders as the primary outcome but not be subjected to the exposure [27, 28, 29]. Accordingly, hospital‐recorded clinical complexity outcomes, which are not subject to PDPM, represent a strong NEDV. In addition, because the average time from hospital discharge to SNF admission is only about 48 h [30], changes in patient condition during this period are minimal (if any) and not a concern for this comparison. Finally, the NEDV design inherently mitigates all patient‐level confounding, given that all patients in the hospital control arm then move to the SNF treatment arm. The difference‐in‐differences analysis with the NEDV control thus estimates the average change in complexity documentation occurring in SNFs attributed to PDPM, above any changes that occurred in the hospital control.

### 2.4 Outcome Measures

The primary outcome in this study was documentation of clinical complexity (i.e., coding intensity), measured in three ways: (1) the number of diagnoses recorded on a claim, (2) weighted Elixhauser score, and (3) the probability of recording five documentation‐sensitive conditions, specifically: chronic pulmonary disease, complicated diabetes, heart failure, obesity, and weight loss. For every hospital‐SNF transition, the unit of analysis, we calculated each clinical complexity outcome first using the hospital claim and again using the SNF claim. The number of diagnoses listed on hospital and SNF claims is a broad measure of clinical complexity. Both hospital and SNF claims allow facilities to record up to 25 diagnoses that are relevant to the care received during the hospital or SNF stay, but only one principal diagnosis is required. The weighted Elixhauser score is a composite measure based on the presence of specific International Classification of Diseases (ICD‐10) diagnoses. It is widely used in studies of patient outcomes and is preferred over the Charlson index in this analysis because it captures a broader range of comorbidities [31]. We used Van Walraven weights, which optimize the score to predict inpatient mortality [32]; scores range from −19 to 89, with higher values indicating greater complexity and mortality risk [33, 34]. Lastly, we identified five documentation‐sensitive conditions a priori as common chronic conditions which could be subject to profit‐motivated coding under PDPM's new incentives because they are included on the NTA component. These conditions are common in the SNF population, typically chronic, and may not require active intervention during a hospital or SNF stay (e.g., we did not select conditions such as multiple sclerosis or AIDS despite their high PDPM reimbursement levels). Therefore, they may be left off a claim if not specifically incentivized. The diagnosis codes used to define these conditions come from the Elixhauser Comorbidity Score ICD‐10 mapping, which are included in Tables S1–S5. Table S6 outlines which NTA categories are related to the selected conditions and the percent of diagnosis codes that overlap with the published NTA lists [10].

### 2.5 Exposures

**FIGURE 1:**
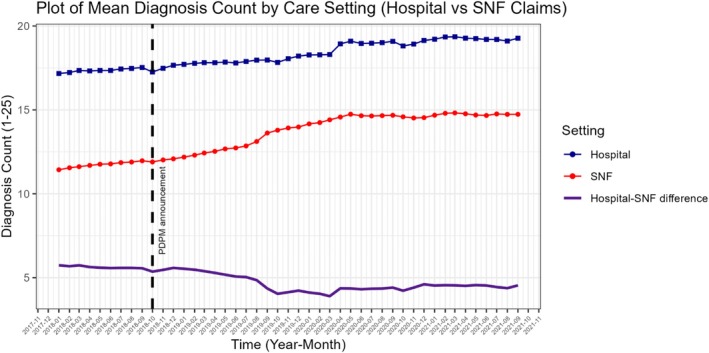
Unadjusted monthly mean diagnosis count among all hospitalized patients (blue line) and those discharged to a skilled nursing facility (SNF) (red line), along with the difference in scores between settings (purple line), before and after the Patient‐Driven Payment Model (PDPM) announcement (vertical dashed line), defined as the Final Rule effective date (October 1, 2018). Time is indexed by the month of hospital admission. A claim can contain up to 25 diagnoses.

**FIGURE 2:**
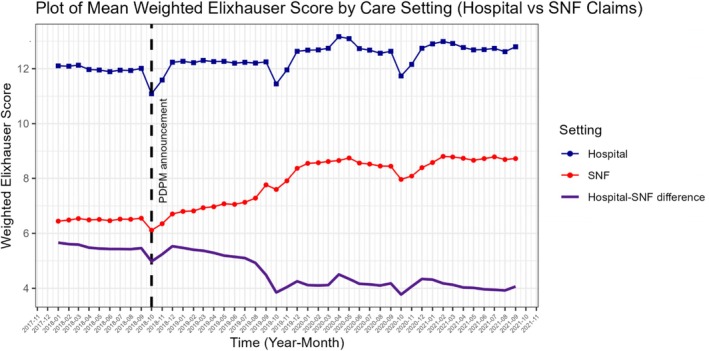
Unadjusted monthly mean weighted Elixhauser scores among all hospitalized patients (blue line) and those discharged to a skilled nursing facility (SNF) (red line), along with the difference in scores between settings (purple line), before and after the Patient‐Driven Payment Model (PDPM) announcement (vertical dashed line), defined as the Final Rule effective date (October 1, 2018). Time is indexed by the month of hospital admission.

The primary exposure in this study was the two‐way interaction between PDPM announcement (defined as the effective date of the Final Rule on October 1, 2018, when the SNF community had ample awareness) and the care setting: hospital or SNF. We selected this date because SNFs started preparing for PDPM—including changing documentation practices—when they became aware of the policy, which has been confirmed in qualitative interviews [35, 36], and is visually apparent in unadjusted plots (Figures 1 and 2 and Figures S1–S7). We also conducted analyses excluding the period of anticipation (October 1, 2018–September 30, 2019), to compare the pre‐PDPM awareness period to the period after which implementation and reimbursement changes officially occurred. The secondary exposure was a three‐way interaction between PDPM announcement, care setting, and SNF profit status (for‐profit vs. not‐for‐profit). SNF profit status was derived from Nursing Home Compare data; not‐for‐profit included both government and faith‐based SNFs.

### 2.6 Confounding Variables

Though all patient level confounding is addressed analytically via the NEDV control, we used data from public files to control for relevant facility‐level factors that could differentially impact documentation practices in each setting [37, 38]. We controlled for hospital participation in accountable care organizations and in the Hospital Value Based Purchasing Program, hospital profit status, hospital rurality (classified using rural–urban commuting area codes linked to hospital zip code as “Isolated rural,” “Small rural,” “Large rural,” and “Urban”), and if the hospital had any affiliated SNFs, per the Provider of Services CMS file, which provides the total number of SNFs a hospital reports being affiliated with, which we dichotomized. We also adjusted for the following SNF factors: SNF profit status (i.e., for‐profit, not‐for‐profit), chain status, overall Nursing Home Compare 5‐Star quality rating, and SNF rurality. Lastly, we attempted to address facility‐level confounding from COVID‐19 by accounting for local COVID‐19 case rates from the county of the hospital or SNF matched to the patient's dates of stay in each respective facility. Case rates were obtained from USAFacts. Other time varying covariates were matched based on the year of facility admission.

### 2.7 Statistical Analysis

We used linear models with time‐ and facility‐based fixed effects and cluster‐robust standard errors (for beneficiary, facility, and time) to estimate changes in three documentation outcomes: (1) number of diagnoses, (2) Elixhauser score, and (3) probability of recording documentation‐sensitive conditions (i.e., chronic pulmonary disease, complicated diabetes, heart failure, obesity, or weight loss). Time effects were defined by the year and month of SNF admission. Primary models used two‐way interaction terms, and secondary models used three‐way interaction terms to quantify variation by SNF profit status. Linear models were used for the continuous outcomes (i.e., number of diagnoses and Elixhauser score). We used generalized linear models (logit link) with the same fixed effect specifications to estimate changes in the five documentation‐sensitive conditions. We report the marginal effects to generate probability‐based estimates, which were very similar to linear probability models [39]. These models are interpreted as the change in probability for the same individual to have a specific condition documented on their SNF claim, before and after PDPM.

#### 2.7.1 Parallel Trends

We assessed the parallel trends assumption through visual inspection of the unadjusted outcome trends, event study plots, and formal joint F‐tests (See Figures S1–S7 for all outcomes). We established statistically relevant parallel trends for all outcomes except for diagnosis count. Diagnosis count still appears visually parallel, but the significant F‐test result may be driven by (i) earlier anticipation affecting diagnosis count before other outcomes given its broad nature, and (ii) the large sample size, which is powered to detect small differences [40]. The magnitude of the post‐policy effect is much larger than any observed deviation in the pre‐policy trend, but results should be interpreted with caution.

All analyses were conducted in RStudio Version 23.06.1, the full model specification is:

$$\text{Yijt}=\beta 0+\beta 1\text{Treatj}+\beta 2\text{Post}+\beta 3\left(\text{Treatj}\times \text{Postt}\right)+\beta 4^{\prime }\mathrm{Xjt}+\mathrm{\alpha j}+\mathrm{\lambda t}+\text{εijt}$$

where $\beta_{0}$ is the intercept, $\beta_{1}$ is the effect of being in the treatment group before PDPM announcement, $\beta_{2}$ is the change after PDPM announcement, $\beta_{3}$ is the DiD effect of PDPM announcement on the treatment group (triply interacted with profit status in DDD models), $X_{\mathrm{jt}}$ is the vector of facility‐level covariates with associated coefficients $\beta_{4}$. $\alpha_{j}$ is the facility fixed effect (SNF or Hospital), $\lambda_{t}$ is the year_month fixed effects relative to SNF admission date, and $\varepsilon_{\mathrm{ijt}}$ is the error term.

## 3 Study Results

### 3.1 Characteristics

Table 1 describes our sample, which included 4,877,166 million hospital‐SNF care episodes with complete data on all covariates and outcomes, representing 3,162,587 million distinct patients over the 45‐month study period (January 2018–October 2021). The majority of SNFs (73%) were for‐profit.

**TABLE 1:** Study outcomes and facility characteristics during study period (*N* = 4,877,166 Hospital‐SNF episodes).

	Pre‐PDPM announcement January 1, 2018–September 30, 2018	Anticipation period October 1, 2018–September 30, 2019	Post‐PDPM October 1, 2019–September 30, 2021	Total (*N* = 4,877,166)
	(*N* = 1,187,989)	(*N* = 1,558,607)	(*N* = 2,130,570)	
*Study outcomes*				
Number of diagnoses (out of 25)				
Mean (SD)	11.7 (6.06)	12.5 (6.23)	14.5 (6.40)	13.2 (6.38)
Median [Min, Max]	11.0 [1.00, 25.0]	12.0 [1.00, 25.0]	14.0 [1.00, 25.0]	12.0 [1.00, 25.0]
Weighted Elixhauser score				
Mean (SD)	6.49 (6.99)	6.89 (7.23)	8.50 (7.94)	7.50 (7.55)
Median [Min, Max]	5.00 [−16.0, 61.0]	5.00 [−16.0, 62.0]	7.00 [−17.0, 63.0]	6.00 [−17.0, 63.0]
Condition prevalence				
Heart failure				
Mean (SD)	0.238 (0.426)	0.251 (0.433)	0.279 (0.449)	0.260 (0.439)
Median [Min, Max]	0 [0, 1.00]	0 [0, 1.00]	0 [0, 1.00]	0 [0, 1.00]
Chronic pulmonary disease				
Mean (SD)	0.223 (0.417)	0.228 (0.420)	0.264 (0.441)	0.243 (0.429)
Median [Min, Max]	0 [0, 1.00]	0 [0, 1.00]	0 [0, 1.00]	0 [0, 1.00]
Complicated diabetes				
Mean (SD)	0.104 (0.305)	0.119 (0.324)	0.164 (0.370)	0.135 (0.342)
Median [Min, Max]	0 [0, 1.00]	0 [0, 1.00]	0 [0, 1.00]	0 [0, 1.00]
Obesity				
Mean (SD)	0.0479 (0.214)	0.0620 (0.241)	0.115 (0.319)	0.0816 (0.274)
Median [Min, Max]	0 [0, 1.00]	0 [0, 1.00]	0 [0, 1.00]	0 [0, 1.00]
Weight loss				
Mean (SD)	0.0380 (0.191)	0.0476 (0.213)	0.136 (0.343)	0.0838 (0.277)
Median [Min, Max]	0 [0, 1.00]	0 [0, 1.00]	0 [0, 1.00]	0 [0, 1.00]
*Hospital characteristics*				
Hospital value‐based purchasing participant	1,087,124 (91.5%)	1,431,997 (91.9%)	1,955,823 (91.8%)	4,474,944 (91.8%)
Hospital ACO participant	10,108 (0.9%)	3743 (0.2%)	7744 (0.4%)	21,595 (0.4%)
For‐profit hospital	178,976 (15.1%)	231,617 (14.9%)	299,458 (14.1%)	710,051 (14.6%)
Hospital with affiliated SNFs	96,811 (8.1%)	128,270 (8.2%)	187,555 (8.8%)	412,636 (8.5%)
Hospital rurality				
Isolated rural	7397 (0.6%)	8618 (0.6%)	10,725 (0.5%)	26,740 (0.5%)
Large rural	118,354 (10.0%)	147,924 (9.5%)	199,445 (9.4%)	465,723 (9.6%)
Small rural	33,321 (2.8%)	40,090 (2.6%)	51,451 (2.4%)	124,862 (2.6%)
Urban	1,026,982 (86.6%)	1,359,517 (87.4%)	1,865,553 (87.7%)	4,252,052 (87.3%)
*SNF characteristics*				
Medicare SNF bed count				
Mean (SD)	10.7 (27.6)	11.3 (28.1)	10.5 (27.1)	10.8 (27.5)
Median [Min, Max]	0 [0, 336]	0 [0, 336]	0 [0, 339]	0 [0, 339]
For‐profit SNF	870,355 (73.3%)	1,131,509 (72.6%)	1,558,532 (73.2%)	3,560,396 (73.0%)
Chain SNF	709,747 (59.7%)	928,936 (59.6%)	1,252,536 (58.8%)	2,891,219 (59.3%)
Overall 5‐star rating				
Mean (SD)	3.54 (1.29)	3.43 (1.27)	3.43 (1.31)	3.46 (1.29)
Median [Min, Max]	3.83 [1.00, 5.00]	3.67 [1.00, 5.00]	3.75 [1.00, 5.00]	3.75 [1.00, 5.00]
SNF rurality				
Isolated rural	27,231 (2.3%)	33,882 (2.2%)	45,611 (2.1%)	106,724 (2.2%)
Large rural	135,809 (11.5%)	171,241 (11.0%)	223,842 (10.5%)	530,892 (10.9%)
Small rural	64,713 (5.5%)	80,177 (5.2%)	104,526 (4.9%)	249,416 (5.1%)
Urban	958,301 (80.8%)	1,270,849 (81.7%)	1,753,195 (82.4%)	3,982,345 (81.8%)

*Note:* Study outcomes and facility characteristics for all hospital‐SNF episodes, stratified into three periods: (1) Pre‐PDPM announcement: January 1, 2018–September 30, 2018, (2) Anticipatory period: October 1, 2018–September 30, 2019, and (3) Post‐PDPM Implementation: October 1, 2019–September 30, 2021.

Abbreviations: ACO, accountable care organization; PDPM, patient‐driven payment model; SNF, skilled nursing facility.

### 3.2 Unadjusted Outcomes

Unadjusted outcomes from SNF claims are presented in Table 1; results are presented across three periods: Pre‐PDPM announcement (January 1, 2018–September 30, 2018), the anticipation period (October 1, 2018–September 30, 2019), and Post‐PDPM implementation (October 1, 2019–September 30, 2021). Figures 1 and 2 also depict the unadjusted monthly mean number of diagnoses and Elixhauser scores on SNF and Hospital claims, as well as the unadjusted difference between settings.

### 3.3 Changes in Clinical Complexity Documentation

Coding intensity changes for the number of diagnoses and Elixhauser scores are presented in Table 2. Full regression results are included in Tables S8–S19. PDPM announcement was associated with an additional 0.83 [95% CI: (0.62, 1.04)] diagnoses above and beyond what would have been expected based on hospital trends. This 0.83 increase in diagnoses represents a relative 7.1% increase as compared to baseline SNF documentation prior to PDPM announcement. PDPM announcement was associated with an additional 0.88 points on the Elixhauser score [95% CI: (0.65, 1.10)] above and beyond what would have been expected, representing a relative 13.6% increase in coding intensity attributed to PDPM. When excluding the period of anticipation, effect sizes increase to a relative 10.6% in diagnosis count and 21.7% on the weighted Elixhauser.

**TABLE 2:** Estimated change in coding intensity associated with PDPM announcement by SNF profit status, including and excluding period of anticipation.

Changes in coding intensity associated with PDPM announcement (*n* = 9,335,052 observations from 4,667,526 hospital‐SNF episodes)				
	Diagnosis count		Weighted Elixhauser	
	Diagnosis count increase; [95% CI]	Relative percent change^a^ (SNF baseline: 11.7)	Point increase; [95% CI]	Relative percent change^a^ (SNF baseline:6.5)
All SNFs	0.83 [0.62, 1.04]	7.1%	0.88; [0.65, 1.10]	13.6%
For‐profit	0.86 [0.63, 1.10]	7.4%	0.92; [0.68, 1.15]	14.1%
Not‐for‐profit	0.70 [0.50, 0.91]	6.0%	0.75 [0.55, 0.96]	11.5%
3‐way interaction term	0.15 [−0.02, 0.31]	1.3%	0.16; [0.06, 0.25]	2.5%

Changes in coding intensity associated with PDPM announcement excluding period of anticipation (*n* = 6,164,774 observations, from 3,082,387 hospital‐SNF episodes)				
	Diagnosis count		Weighted Elixhauser	
	Diagnosis count increase; [95% CI]	Relative percent change (SNF baseline: 11.7)	Point increase; [95% CI]	Relative percent change (SNF baseline:6.5)
All SNFs	1.24 [1.07, 1.41]	10.6%	1.41 [1.30, 1.52]	21.7%
For‐profit	1.33 [1.15, 1.51]	11.4%	1.50 [1.38, 1.61]	23.1%
Not‐for‐profit	1.00 [0.77, 1.24]	8.5%	1.23 [1.09, 1.37]	18.9%
3‐way interaction term	0.33 [0.11, 0.55]	2.8%	0.26 [0.13, 0.39]	4.0%

*Note:* Difference‐in‐difference estimates of changes in the number of diagnoses and weighted Elixhauser score among for‐profit and not‐for‐profit SNFs, with a three‐way interaction term (Post‐PDPM × care setting × profit status). Section 1 includes the full study period; Section 2 excludes the anticipatory period (October 1, 2018–September 30, 2019).

Abbreviations: PDPM, patient‐driven payment model; SNF, skilled nursing facility.

^a^ Relative percent increase reflects the additional change compared with the unadjusted pre‐PDPM baseline for each model; for the three‐way interaction, it reflects the additional relative increase in for‐profit facilities compared with non‐profits.

Results for the five documentation‐sensitive conditions are in Table 3. There was a significant increase in the probability of documentation for all five conditions after PDPM announcement. From smallest to largest increases, we observed a 2.80 percentage point [95% CI: 2.1, 3.4], or 11.7% relative increase in the documentation of heart failure, a 3.9 percentage point [95% CI: 3.0, 4.9], or 17.5% relative increase in the documentation of chronic pulmonary disease, a 5.0 percentage point [95% CI: 3.9, 6.1], or 48.0% relative increase in the documentation of complicated diabetes, a 7.3 percentage point [95% CI: 6.0, 8.9], or 152% relative increase in the documentation of obesity, and a 9.8 percentage point [95% CI: 7.4, 12.2], or 257% relative increase in the documentation of weight loss. In all models, the relative increases are calculated as the estimated additional effect divided by the pre‐period SNF baseline level.

**TABLE 3:** Estimated change in the probability of recording five documentation‐sensitive conditions.

Condition	Percentage point increase; [95% CI]	Relative percent increase^a^
Chronic pulmonary disease	3.9 pp; [3.0, 4.9]	17.5%
Complicated diabetes	5.0 pp; [3.9, 6.1]	48%
Heart failure	2.8 pp; [2.1, 3.4]	11.7%
Obesity	7.3 pp; [6.0, 8.9]	152%
Weight loss	9.8 pp; [7.4, 12.2]	257%

*Note:* Difference‐in‐difference estimation averages for the change in probability of documentation (marginal effect estimation), before and after patient‐driven payment model announcement (October 1, 2018), for each of the five documentation‐sensitive conditions. All *p* < 0.001.

^a^ Relative percent increase represents the additional increase as compared to the unadjusted baseline in the pre‐period for each model.

### 3.4 Variability by SNF Profit Status

In secondary triple interaction models, we observed significant differential effects of PDPM announcement on both Elixhauser score and diagnosis count based on SNF profit status. The differential effect of PDPM announcement in for‐profit SNFs was 0.16 additional Elixhauser points [95% CI: (0.06, 0.25)] compared to not‐for‐profit SNFs, representing a relative increase of 2.5%. We estimate an additional 0.15 diagnoses in for‐profit SNFs [95% CI: (−0.02, 0.31)], representing a 1.3% increase, but this was not statistically significant (*p* = 0.08). The effect sizes on triple interaction terms increased when estimating the effect of PDPM excluding the period of anticipation, to a relative 4% on the Elixhauser and 2.8% on diagnosis count, both with *p* < 0.001. To complement these findings, we also ran stratum‐specific models within for‐profit and not‐for‐profit subsamples (without the profit interaction term), allowing direct comparison of effect sizes across ownership types, which are all shown in Table 2.

## 4 Discussion

In this quasi‐experimental study examining the impact of PDPM on coding intensity in SNF claims compared to hospital claim controls, we found evidence of increased complexity documentation for all SNFs above and beyond what would be expected had PDPM not occurred. Increased documentation of clinical complexity was more prevalent among for‐profit SNFs, for both the total number of diagnoses on SNF claims and the Elixhauser score. Consistent with our hypotheses, we also observed relatively large increases in the probability of recording five documentation‐sensitive conditions. These findings are consistent with prior literature on SNFs' responses to payment incentives [21, 22]. CMS frequently adjusts payments (prospectively or retroactively) to account for anticipated changes in facility behavior in response to financial incentives [41, 42, 43, 44]. These behavioral adjustments are intended to address early shifts in documentation or service use that deviate from the policy's intent and are typically based on historical data from prior models [44]. When initially proposed, PDPM did not account for proactive behavioral adjustments, but rather relied on a retroactive payment parity adjustment to account for behavioral changes [45]. The retroactive adjustment relied on the RUG‐IV case mix distribution as a proxy for what would have occurred in the absence of PDPM [41, 42, 46]. In contrast, our analysis uses a much stronger counterfactual, enabling a more accurate estimate of PDPM's effect on documentation changes. This design strengthens the evidence base to inform behavioral adjustments and planning for Medicare financial sustainability.

Our findings on differential effects based on SNF profit status also have important implications beyond payment adjustment. Facility‐level measures of patient complexity factor heavily into SNF performance evaluation in the Medicare quality reporting and value‐based purchasing programs [47, 48]. Though the reasons for our differential findings may be multifactorial, the “one size fits all” approach to SNF payment policy currently ignores the nuance that different types of SNFs may react to financial policy differently [22]. Comparison of Table 2 models, including vs. excluding the period of anticipation, suggests that variation by profit status became more significant when formal reimbursement changes occurred in October 2019. Our results indicate that facility‐level factors, including profit status, may be important to consider in policy refinement, payment adjustment, as well as in SNF monitoring and auditing efforts.

In addition, our analysis underscores the importance of using hospital‐level claims rather than SNF claims to measure clinical complexity in SNF‐related longitudinal research that spans PDPM implementation. Our findings demonstrate that SNF‐derived complexity and comorbidity measurements from administrative data are not comparable for the same patients before and after October 2019. Accordingly, SNF research incorporating patient‐level complexity measures before and after 2019 should utilize hospital‐derived complexity. Similarly, from a policy perspective, risk‐adjusted quality measures from this period should also avoid using SNF measurement, which may not have been initially apparent before considering the impact of PDPM [23].

Our examination of documentation‐sensitive conditions offers insight into how SNFs may have responded to PDPM's incentives. While the PDPM algorithm is complex, it is not fully opaque—CMS publicly specifies the NTA component, including a list of prioritized conditions and services tied to higher reimbursement. While our selected conditions were not operationalized specifically using published lists of diagnosis codes on the NTA list, we still observed large relative increases in documentation across all five conditions studied, regardless of how strongly they were prioritized in the NTA component. This suggests SNFs may have implemented broad documentation changes rather than targeting only high‐priority diagnoses. Notably, none of the Elixhauser weight loss codes are prioritized under PDPM, yet weight loss saw the largest increase in documentation (9.8 pp); this likely reflects existing weight loss in the SNF community, which was documented at very low rates prior to PDPM. The change in obesity (7.3 pp) may reflect something similar. In addition, weight loss is tied to services like parenteral feeding and IV‐fluid administration, both of which are highly prioritized under the NTA component. It is possible that increased awareness of the condition also led to significant increases in documentation.

While our design provided a precise estimate of changes in documentation after PDPM, without a measure of SNF complexity that fully is unrelated to PDPM, it is not possible to precisely disentangle whether the increases in complexity documentation reflect problematic upcoding. Future work that builds upon our identified subgroup differences may improve efforts to disentangle problematic versus appropriate coding changes. As a first step, this work adds to the discussion about potentially profit‐driven changes in documentation practices after PDPM [49]. We found that for‐profit SNFs are more responsive to PDPM than not‐for‐profits, which is consistent with findings from other studies on profit‐motivated behavior changes [49, 50, 51, 52]. Coding intensity did increase for all SNFs regardless of profit‐status, however the magnitude of for‐profit vs. not‐for‐profit differences (roughly 2%) may translate to substantial shifts in payment in a $25 billion dollar industry. Further investigation of these differences is warranted. If SNF behavioral responses to policy changes vary by facility characteristics, such as profit status, targeted monitoring and auditing may be warranted.

Finally, the SNF industry was aware of PDPM prior to its implementation, given the rulemaking process and public guidance issued over a year in advance. Qualitative research confirms that many SNFs began preparing for PDPM before the official start date, including operational changes and increased administrative awareness of new documentation guidance [35, 36]. Accordingly, we present results anchored to the effective date of the Final Rule, both including and excluding the period of anticipation, differentiating our work from recent research exploring a similar question using a regression discontinuity design. The overall conclusions of both studies are similar [52, 53].

### 4.1 Limitations

The use of administrative data is essential to answering our research questions, but it limits our ability to capture more nuanced dimensions of clinical complexity outside of documented diagnoses where qualitative data may provide deeper insight. Additionally, we cannot disentangle strategic upcoding from improved, accurate documentation, which is a common limitation in most studies on documentation patterns [19, 20, 54]. The NEDV design helps address patient‐centric COVID‐related confounding, particularly changes in patient complexity, by comparing within‐beneficiary observations. Though we are unable to fully address unobserved facility‐level confounding in setting‐specific responses to COVID‐19 that affected coding intensity, we do use county‐level COVID‐19 case rates tied to each hospital or SNF to address some of this potential confounding. Finally, if the parallel trends assumption is not perfectly met (i.e., for diagnosis count), which cannot ever be verified, results should be understood as associations without causal underpinnings.

## 5 Conclusion

Our novel analysis of Medicare claims data using within‐patient episodes identified evidence of potentially profit‐motivated changes in coding intensity tied to PDPM. We observe increased coding intensity, particularly among for‐profit SNFs, along with notable rises in the probability of recording documentation‐sensitive conditions. Some of these behaviors may reflect a more accurate documentation of existing complexity, which aligns with the intent of PDPM to support care for clinically complex patients. However, if certain facilities are inflating clinical complexity rather than reflecting true medical needs, Medicare may be overpaying, straining a program that millions depend on. Yet, if uniform corrective measures are applied to address potentially problematic upcoding, certain facilities may be inadvertently penalized, hindering their ability to care for existing complex patients. These findings may help guide evidence‐based refinements to SNF payment policy and support targeted monitoring or auditing strategies that account for differences across facility type.

## Funding

This study was supported with funding from the National Institute on Aging (AG065371). Research reported in this publication was also supported by the National Center for Advancing Translational Sciences of the National Institutes of Health under award number TL1TR002318 (Amaravadi). Support for data access and analyses for this research came from the University of Washington (UW)'s Population Health Initiative, UW's Student Technology Fee program, the UW's Provost's office, and a Eunice Kennedy Shriver National Institute of Child Health and Human Development research infrastructure grant, P2C HD042828, to the Center for Studies in Demography & Ecology at the University of Washington. The content is solely the responsibility of the authors and does not necessarily represent the official views of the National Institutes of Health.

## Conflicts of Interest

The authors declare no conflicts of interest.

## Supporting information


**Data S1:** Supporting Information.

## Data Availability

The data that support the findings of this study are available from the Centers for Medicare and Medicaid Services. Restrictions apply to the availability of these data, which were used under license for this study. Data are available from https://resdac.org/ with the permission of the Centers for Medicare and Medicaid Services.
