# Supplementary material for: Too Sick to be True? Evaluating Potentially Problematic Diagnosis Coding Practices in Medicare's Patient‐Driven Payment Model
Source: Health Serv Res. 2026 Feb 9;61(2):e70084. doi: 10.1111/1475-6773.70084 (PMC12884731; doi:10.1111/1475-6773.70084)
Supplement: Supplementary file 1 — Data S1: Supporting Information. [file HESR-61-e70084-s001.docx]

# Supplementary Materials (Tables & Figures)

## Table S1. ICD- 10 Codes Used to Define Chronic Pulmonary Disease (N = 68)

| **ICD- 10 Codes Used to Define Chronic Pulmonary Disease** (n = 68) | |
| --- | --- |
| J410 | Simple chronic bronchitis |
| J411 | Mucopurulent chronic bronchitis |
| J418 | Mixed simple and mucopurulent chronic bronchitis |
| J42 | Unspecified chronic bronchitis |
| J430 | Unilateral pulmonary emphysema [MacLeod's syndrome] |
| J431 | Panlobular emphysema |
| J432 | Centrilobular emphysema |
| J438 | Other emphysema |
| J439 | Emphysema, unspecified |
| J440 | Chronic obstructive pulmonary disease with (acute) lower respiratory infection |
| J441 | Chronic obstructive pulmonary disease with (acute) exacerbation |
| J4481 | Bronchiolitis obliterans and bronchiolitis obliterans syndrome |
| J4489 | Other specified chronic obstructive pulmonary disease |
| J449 | Chronic obstructive pulmonary disease, unspecified |
| J4520 | Mild intermittent asthma, uncomplicated |
| J4521 | Mild intermittent asthma with (acute) exacerbation |
| J4522 | Mild intermittent asthma with status asthmaticus |
| J4530 | Mild persistent asthma, uncomplicated |
| J4531 | Mild persistent asthma with (acute) exacerbation |
| J4532 | Mild persistent asthma with status asthmaticus |
| J4540 | Moderate persistent asthma, uncomplicated |
| J4541 | Moderate persistent asthma with (acute) exacerbation |
| J4542 | Moderate persistent asthma with status asthmaticus |
| J4550 | Severe persistent asthma, uncomplicated |
| J4551 | Severe persistent asthma with (acute) exacerbation |
| J4552 | Severe persistent asthma with status asthmaticus |
| J45901 | Unspecified asthma with (acute) exacerbation |
| J45902 | Unspecified asthma with status asthmaticus |
| J45909 | Unspecified asthma, uncomplicated |
| J45990 | Exercise induced bronchospasm |
| J45991 | Cough variant asthma |
| J45998 | Other asthma |
| J470 | Bronchiectasis with acute lower respiratory infection |
| J471 | Bronchiectasis with (acute) exacerbation |
| J479 | Bronchiectasis, uncomplicated |
| J4A0 | Restrictive allograft syndrome |
| J4A8 | Other chronic lung allograft dysfunction |
| J4A9 | Chronic lung allograft dysfunction, unspecified |
| J60 | Coalworker's pneumoconiosis |
| J61 | Pneumoconiosis due to asbestos and other mineral fibers |
| J620 | Pneumoconiosis due to talc dust |
| J628 | Pneumoconiosis due to other dust containing silica |
| J630 | Aluminosis (of lung) |
| J631 | Bauxite fibrosis (of lung) |
| J632 | Berylliosis |
| J633 | Graphite fibrosis (of lung) |
| J634 | Siderosis |
| J635 | Stannosis |
| J636 | Pneumoconiosis due to other specified inorganic dusts |
| J64 | Unspecified pneumoconiosis |
| J65 | Pneumoconiosis associated with tuberculosis |
| J660 | Byssinosis |
| J661 | Flax-dressers' disease |
| J662 | Cannabinosis |
| J668 | Airway disease due to other specific organic dusts |
| J670 | Farmer's lung |
| J671 | Bagassosis |
| J672 | Bird fancier's lung |
| J673 | Suberosis |
| J674 | Maltworker's lung |
| J675 | Mushroom-worker's lung |
| J676 | Maple-bark-stripper's lung |
| J677 | Air conditioner and humidifier lung |
| J678 | Hypersensitivity pneumonitis due to other organic dusts |
| J679 | Hypersensitivity pneumonitis due to unspecified organic dust |
| J684 | Chronic respiratory conditions due to chemicals, gases, fumes and vapors |
| J701 | Chronic and other pulmonary manifestations due to radiation |
| J703 | Chronic drug-induced interstitial lung disorders |

## Table S2. ICD- 10 Codes Used to Define Complicated Diabetes (N = 445)

| **ICD- 10 Codes Used to Define Complicated Diabetes** (n = 445) | |
| --- | --- |
| E0821 | Diabetes mellitus due to underlying condition with diabetic nephropathy |
| E0822 | Diabetes mellitus due to underlying condition with diabetic chronic kidney disease |
| E0829 | Diabetes mellitus due to underlying condition with other diabetic kidney complication |
| E08311 | Diabetes mellitus due to underlying condition with unspecified diabetic retinopathy with macular edema |
| E08319 | Diabetes mellitus due to underlying condition with unspecified diabetic retinopathy without macular edema |
| E08321 | Diabetes mellitus due to underlying condition with mild nonproliferative diabetic retinopathy with macular edema |
| E083211 | Diabetes mellitus due to underlying condition with mild nonproliferative diabetic retinopathy with macular edema, right eye |
| E083212 | Diabetes mellitus due to underlying condition with mild nonproliferative diabetic retinopathy with macular edema, left eye |
| E083213 | Diabetes mellitus due to underlying condition with mild nonproliferative diabetic retinopathy with macular edema, bilateral |
| E083219 | Diabetes mellitus due to underlying condition with mild nonproliferative diabetic retinopathy with macular edema, unspecified eye |
| E08329 | Diabetes mellitus due to underlying condition with mild nonproliferative diabetic retinopathy without macular edema |
| E083291 | Diabetes mellitus due to underlying condition with mild nonproliferative diabetic retinopathy without macular edema, right eye |
| E083292 | Diabetes mellitus due to underlying condition with mild nonproliferative diabetic retinopathy without macular edema, left eye |
| E083293 | Diabetes mellitus due to underlying condition with mild nonproliferative diabetic retinopathy without macular edema, bilateral |
| E083299 | Diabetes mellitus due to underlying condition with mild nonproliferative diabetic retinopathy without macular edema, unspecified eye |
| E08331 | Diabetes mellitus due to underlying condition with moderate nonproliferative diabetic retinopathy with macular edema |
| E083311 | Diabetes mellitus due to underlying condition with moderate nonproliferative diabetic retinopathy with macular edema, right eye |
| E083312 | Diabetes mellitus due to underlying condition with moderate nonproliferative diabetic retinopathy with macular edema, left eye |
| E083313 | Diabetes mellitus due to underlying condition with moderate nonproliferative diabetic retinopathy with macular edema, bilateral |
| E083319 | Diabetes mellitus due to underlying condition with moderate nonproliferative diabetic retinopathy with macular edema, unspecified eye |
| E08339 | Diabetes mellitus due to underlying condition with moderate nonproliferative diabetic retinopathy without macular edema |
| E083391 | Diabetes mellitus due to underlying condition with moderate nonproliferative diabetic retinopathy without macular edema, right eye |
| E083392 | Diabetes mellitus due to underlying condition with moderate nonproliferative diabetic retinopathy without macular edema, left eye |
| E083393 | Diabetes mellitus due to underlying condition with moderate nonproliferative diabetic retinopathy without macular edema, bilateral |
| E083399 | Diabetes mellitus due to underlying condition with moderate nonproliferative diabetic retinopathy without macular edema, unspecified eye |
| E08341 | Diabetes mellitus due to underlying condition with severe nonproliferative diabetic retinopathy with macular edema |
| E083411 | Diabetes mellitus due to underlying condition with severe nonproliferative diabetic retinopathy with macular edema, right eye |
| E083412 | Diabetes mellitus due to underlying condition with severe nonproliferative diabetic retinopathy with macular edema, left eye |
| E083413 | Diabetes mellitus due to underlying condition with severe nonproliferative diabetic retinopathy with macular edema, bilateral |
| E083419 | Diabetes mellitus due to underlying condition with severe nonproliferative diabetic retinopathy with macular edema, unspecified eye |
| E08349 | Diabetes mellitus due to underlying condition with severe nonproliferative diabetic retinopathy without macular edema |
| E083491 | Diabetes mellitus due to underlying condition with severe nonproliferative diabetic retinopathy without macular edema, right eye |
| E083492 | Diabetes mellitus due to underlying condition with severe nonproliferative diabetic retinopathy without macular edema, left eye |
| E083493 | Diabetes mellitus due to underlying condition with severe nonproliferative diabetic retinopathy without macular edema, bilateral |
| E083499 | Diabetes mellitus due to underlying condition with severe nonproliferative diabetic retinopathy without macular edema, unspecified eye |
| E08351 | Diabetes mellitus due to underlying condition with proliferative diabetic retinopathy with macular edema |
| E083511 | Diabetes mellitus due to underlying condition with proliferative diabetic retinopathy with macular edema, right eye |
| E083512 | Diabetes mellitus due to underlying condition with proliferative diabetic retinopathy with macular edema, left eye |
| E083513 | Diabetes mellitus due to underlying condition with proliferative diabetic retinopathy with macular edema, bilateral |
| E083519 | Diabetes mellitus due to underlying condition with proliferative diabetic retinopathy with macular edema, unspecified eye |
| E083521 | Diabetes mellitus due to underlying condition with proliferative diabetic retinopathy with traction retinal detachment involving the macula, right eye |
| E083522 | Diabetes mellitus due to underlying condition with proliferative diabetic retinopathy with traction retinal detachment involving the macula, left eye |
| E083523 | Diabetes mellitus due to underlying condition with proliferative diabetic retinopathy with traction retinal detachment involving the macula, bilateral |
| E083529 | Diabetes mellitus due to underlying condition with proliferative diabetic retinopathy with traction retinal detachment involving the macula, unspecified eye |
| E083531 | Diabetes mellitus due to underlying condition with proliferative diabetic retinopathy with traction retinal detachment not involving the macula, right eye |
| E083532 | Diabetes mellitus due to underlying condition with proliferative diabetic retinopathy with traction retinal detachment not involving the macula, left eye |
| E083533 | Diabetes mellitus due to underlying condition with proliferative diabetic retinopathy with traction retinal detachment not involving the macula, bilateral |
| E083539 | Diabetes mellitus due to underlying condition with proliferative diabetic retinopathy with traction retinal detachment not involving the macula, unspecified eye |
| E083541 | Diabetes mellitus due to underlying condition with proliferative diabetic retinopathy with combined traction retinal detachment and rhegmatogenous retinal detachment, right eye |
| E083542 | Diabetes mellitus due to underlying condition with proliferative diabetic retinopathy with combined traction retinal detachment and rhegmatogenous retinal detachment, left eye |
| E083543 | Diabetes mellitus due to underlying condition with proliferative diabetic retinopathy with combined traction retinal detachment and rhegmatogenous retinal detachment, bilateral |
| E083549 | Diabetes mellitus due to underlying condition with proliferative diabetic retinopathy with combined traction retinal detachment and rhegmatogenous retinal detachment, unspecified eye |
| E083551 | Diabetes mellitus due to underlying condition with stable proliferative diabetic retinopathy, right eye |
| E083552 | Diabetes mellitus due to underlying condition with stable proliferative diabetic retinopathy, left eye |
| E083553 | Diabetes mellitus due to underlying condition with stable proliferative diabetic retinopathy, bilateral |
| E083559 | Diabetes mellitus due to underlying condition with stable proliferative diabetic retinopathy, unspecified eye |
| E08359 | Diabetes mellitus due to underlying condition with proliferative diabetic retinopathy without macular edema |
| E083591 | Diabetes mellitus due to underlying condition with proliferative diabetic retinopathy without macular edema, right eye |
| E083592 | Diabetes mellitus due to underlying condition with proliferative diabetic retinopathy without macular edema, left eye |
| E083593 | Diabetes mellitus due to underlying condition with proliferative diabetic retinopathy without macular edema, bilateral |
| E083599 | Diabetes mellitus due to underlying condition with proliferative diabetic retinopathy without macular edema, unspecified eye |
| E0836 | Diabetes mellitus due to underlying condition with diabetic cataract |
| E0837X1 | Diabetes mellitus due to underlying condition with diabetic macular edema, resolved following treatment, right eye |
| E0837X2 | Diabetes mellitus due to underlying condition with diabetic macular edema, resolved following treatment, left eye |
| E0837X3 | Diabetes mellitus due to underlying condition with diabetic macular edema, resolved following treatment, bilateral |
| E0837X9 | Diabetes mellitus due to underlying condition with diabetic macular edema, resolved following treatment, unspecified eye |
| E0839 | Diabetes mellitus due to underlying condition with other diabetic ophthalmic complication |
| E0840 | Diabetes mellitus due to underlying condition with diabetic neuropathy, unspecified |
| E0841 | Diabetes mellitus due to underlying condition with diabetic mononeuropathy |
| E0842 | Diabetes mellitus due to underlying condition with diabetic polyneuropathy |
| E0843 | Diabetes mellitus due to underlying condition with diabetic autonomic (poly)neuropathy |
| E0844 | Diabetes mellitus due to underlying condition with diabetic amyotrophy |
| E0849 | Diabetes mellitus due to underlying condition with other diabetic neurological complication |
| E0851 | Diabetes mellitus due to underlying condition with diabetic peripheral angiopathy without gangrene |
| E0852 | Diabetes mellitus due to underlying condition with diabetic peripheral angiopathy with gangrene |
| E0859 | Diabetes mellitus due to underlying condition with other circulatory complications |
| E08610 | Diabetes mellitus due to underlying condition with diabetic neuropathic arthropathy |
| E08618 | Diabetes mellitus due to underlying condition with other diabetic arthropathy |
| E08620 | Diabetes mellitus due to underlying condition with diabetic dermatitis |
| E08621 | Diabetes mellitus due to underlying condition with foot ulcer |
| E08622 | Diabetes mellitus due to underlying condition with other skin ulcer |
| E08628 | Diabetes mellitus due to underlying condition with other skin complications |
| E08630 | Diabetes mellitus due to underlying condition with periodontal disease |
| E08638 | Diabetes mellitus due to underlying condition with other oral complications |
| E08641 | Diabetes mellitus due to underlying condition with hypoglycemia with coma |
| E08649 | Diabetes mellitus due to underlying condition with hypoglycemia without coma |
| E0865 | Diabetes mellitus due to underlying condition with hyperglycemia |
| E0869 | Diabetes mellitus due to underlying condition with other specified complication |
| E088 | Diabetes mellitus due to underlying condition with unspecified complications |
| E0921 | Drug or chemical induced diabetes mellitus with diabetic nephropathy |
| E0922 | Drug or chemical induced diabetes mellitus with diabetic chronic kidney disease |
| E0929 | Drug or chemical induced diabetes mellitus with other diabetic kidney complication |
| E09311 | Drug or chemical induced diabetes mellitus with unspecified diabetic retinopathy with macular edema |
| E09319 | Drug or chemical induced diabetes mellitus with unspecified diabetic retinopathy without macular edema |
| E09321 | Drug or chemical induced diabetes mellitus with mild nonproliferative diabetic retinopathy with macular edema |
| E093211 | Drug or chemical induced diabetes mellitus with mild nonproliferative diabetic retinopathy with macular edema, right eye |
| E093212 | Drug or chemical induced diabetes mellitus with mild nonproliferative diabetic retinopathy with macular edema, left eye |
| E093213 | Drug or chemical induced diabetes mellitus with mild nonproliferative diabetic retinopathy with macular edema, bilateral |
| E093219 | Drug or chemical induced diabetes mellitus with mild nonproliferative diabetic retinopathy with macular edema, unspecified eye |
| E09329 | Drug or chemical induced diabetes mellitus with mild nonproliferative diabetic retinopathy without macular edema |
| E093291 | Drug or chemical induced diabetes mellitus with mild nonproliferative diabetic retinopathy without macular edema, right eye |
| E093292 | Drug or chemical induced diabetes mellitus with mild nonproliferative diabetic retinopathy without macular edema, left eye |
| E093293 | Drug or chemical induced diabetes mellitus with mild nonproliferative diabetic retinopathy without macular edema, bilateral |
| E093299 | Drug or chemical induced diabetes mellitus with mild nonproliferative diabetic retinopathy without macular edema, unspecified eye |
| E09331 | Drug or chemical induced diabetes mellitus with moderate nonproliferative diabetic retinopathy with macular edema |
| E093311 | Drug or chemical induced diabetes mellitus with moderate nonproliferative diabetic retinopathy with macular edema, right eye |
| E093312 | Drug or chemical induced diabetes mellitus with moderate nonproliferative diabetic retinopathy with macular edema, left eye |
| E093313 | Drug or chemical induced diabetes mellitus with moderate nonproliferative diabetic retinopathy with macular edema, bilateral |
| E093319 | Drug or chemical induced diabetes mellitus with moderate nonproliferative diabetic retinopathy with macular edema, unspecified eye |
| E09339 | Drug or chemical induced diabetes mellitus with moderate nonproliferative diabetic retinopathy without macular edema |
| E093391 | Drug or chemical induced diabetes mellitus with moderate nonproliferative diabetic retinopathy without macular edema, right eye |
| E093392 | Drug or chemical induced diabetes mellitus with moderate nonproliferative diabetic retinopathy without macular edema, left eye |
| E093393 | Drug or chemical induced diabetes mellitus with moderate nonproliferative diabetic retinopathy without macular edema, bilateral |
| E093399 | Drug or chemical induced diabetes mellitus with moderate nonproliferative diabetic retinopathy without macular edema, unspecified eye |
| E09341 | Drug or chemical induced diabetes mellitus with severe nonproliferative diabetic retinopathy with macular edema |
| E093411 | Drug or chemical induced diabetes mellitus with severe nonproliferative diabetic retinopathy with macular edema, right eye |
| E093412 | Drug or chemical induced diabetes mellitus with severe nonproliferative diabetic retinopathy with macular edema, left eye |
| E093413 | Drug or chemical induced diabetes mellitus with severe nonproliferative diabetic retinopathy with macular edema, bilateral |
| E093419 | Drug or chemical induced diabetes mellitus with severe nonproliferative diabetic retinopathy with macular edema, unspecified eye |
| E09349 | Drug or chemical induced diabetes mellitus with severe nonproliferative diabetic retinopathy without macular edema |
| E093491 | Drug or chemical induced diabetes mellitus with severe nonproliferative diabetic retinopathy without macular edema, right eye |
| E093492 | Drug or chemical induced diabetes mellitus with severe nonproliferative diabetic retinopathy without macular edema, left eye |
| E093493 | Drug or chemical induced diabetes mellitus with severe nonproliferative diabetic retinopathy without macular edema, bilateral |
| E093499 | Drug or chemical induced diabetes mellitus with severe nonproliferative diabetic retinopathy without macular edema, unspecified eye |
| E09351 | Drug or chemical induced diabetes mellitus with proliferative diabetic retinopathy with macular edema |
| E093511 | Drug or chemical induced diabetes mellitus with proliferative diabetic retinopathy with macular edema, right eye |
| E093512 | Drug or chemical induced diabetes mellitus with proliferative diabetic retinopathy with macular edema, left eye |
| E093513 | Drug or chemical induced diabetes mellitus with proliferative diabetic retinopathy with macular edema, bilateral |
| E093519 | Drug or chemical induced diabetes mellitus with proliferative diabetic retinopathy with macular edema, unspecified eye |
| E093521 | Drug or chemical induced diabetes mellitus with proliferative diabetic retinopathy with traction retinal detachment involving the macula, right eye |
| E093522 | Drug or chemical induced diabetes mellitus with proliferative diabetic retinopathy with traction retinal detachment involving the macula, left eye |
| E093523 | Drug or chemical induced diabetes mellitus with proliferative diabetic retinopathy with traction retinal detachment involving the macula, bilateral |
| E093529 | Drug or chemical induced diabetes mellitus with proliferative diabetic retinopathy with traction retinal detachment involving the macula, unspecified eye |
| E093531 | Drug or chemical induced diabetes mellitus with proliferative diabetic retinopathy with traction retinal detachment not involving the macula, right eye |
| E093532 | Drug or chemical induced diabetes mellitus with proliferative diabetic retinopathy with traction retinal detachment not involving the macula, left eye |
| E093533 | Drug or chemical induced diabetes mellitus with proliferative diabetic retinopathy with traction retinal detachment not involving the macula, bilateral |
| E093539 | Drug or chemical induced diabetes mellitus with proliferative diabetic retinopathy with traction retinal detachment not involving the macula, unspecified eye |
| E093541 | Drug or chemical induced diabetes mellitus with proliferative diabetic retinopathy with combined traction retinal detachment and rhegmatogenous retinal detachment, right eye |
| E093542 | Drug or chemical induced diabetes mellitus with proliferative diabetic retinopathy with combined traction retinal detachment and rhegmatogenous retinal detachment, left eye |
| E093543 | Drug or chemical induced diabetes mellitus with proliferative diabetic retinopathy with combined traction retinal detachment and rhegmatogenous retinal detachment, bilateral |
| E093549 | Drug or chemical induced diabetes mellitus with proliferative diabetic retinopathy with combined traction retinal detachment and rhegmatogenous retinal detachment, unspecified eye |
| E093551 | Drug or chemical induced diabetes mellitus with stable proliferative diabetic retinopathy, right eye |
| E093552 | Drug or chemical induced diabetes mellitus with stable proliferative diabetic retinopathy, left eye |
| E093553 | Drug or chemical induced diabetes mellitus with stable proliferative diabetic retinopathy, bilateral |
| E093559 | Drug or chemical induced diabetes mellitus with stable proliferative diabetic retinopathy, unspecified eye |
| E09359 | Drug or chemical induced diabetes mellitus with proliferative diabetic retinopathy without macular edema |
| E093591 | Drug or chemical induced diabetes mellitus with proliferative diabetic retinopathy without macular edema, right eye |
| E093592 | Drug or chemical induced diabetes mellitus with proliferative diabetic retinopathy without macular edema, left eye |
| E093593 | Drug or chemical induced diabetes mellitus with proliferative diabetic retinopathy without macular edema, bilateral |
| E093599 | Drug or chemical induced diabetes mellitus with proliferative diabetic retinopathy without macular edema, unspecified eye |
| E0936 | Drug or chemical induced diabetes mellitus with diabetic cataract |
| E0937X1 | Drug or chemical induced diabetes mellitus with diabetic macular edema, resolved following treatment, right eye |
| E0937X2 | Drug or chemical induced diabetes mellitus with diabetic macular edema, resolved following treatment, left eye |
| E0937X3 | Drug or chemical induced diabetes mellitus with diabetic macular edema, resolved following treatment, bilateral |
| E0937X9 | Drug or chemical induced diabetes mellitus with diabetic macular edema, resolved following treatment, unspecified eye |
| E0939 | Drug or chemical induced diabetes mellitus with other diabetic ophthalmic complication |
| E0940 | Drug or chemical induced diabetes mellitus with neurological complications with diabetic neuropathy, unspecified |
| E0941 | Drug or chemical induced diabetes mellitus with neurological complications with diabetic mononeuropathy |
| E0942 | Drug or chemical induced diabetes mellitus with neurological complications with diabetic polyneuropathy |
| E0943 | Drug or chemical induced diabetes mellitus with neurological complications with diabetic autonomic (poly)neuropathy |
| E0944 | Drug or chemical induced diabetes mellitus with neurological complications with diabetic amyotrophy |
| E0949 | Drug or chemical induced diabetes mellitus with neurological complications with other diabetic neurological complication |
| E0951 | Drug or chemical induced diabetes mellitus with diabetic peripheral angiopathy without gangrene |
| E0952 | Drug or chemical induced diabetes mellitus with diabetic peripheral angiopathy with gangrene |
| E0959 | Drug or chemical induced diabetes mellitus with other circulatory complications |
| E09610 | Drug or chemical induced diabetes mellitus with diabetic neuropathic arthropathy |
| E09618 | Drug or chemical induced diabetes mellitus with other diabetic arthropathy |
| E09620 | Drug or chemical induced diabetes mellitus with diabetic dermatitis |
| E09621 | Drug or chemical induced diabetes mellitus with foot ulcer |
| E09622 | Drug or chemical induced diabetes mellitus with other skin ulcer |
| E09628 | Drug or chemical induced diabetes mellitus with other skin complications |
| E09630 | Drug or chemical induced diabetes mellitus with periodontal disease |
| E09638 | Drug or chemical induced diabetes mellitus with other oral complications |
| E09641 | Drug or chemical induced diabetes mellitus with hypoglycemia with coma |
| E09649 | Drug or chemical induced diabetes mellitus with hypoglycemia without coma |
| E0965 | Drug or chemical induced diabetes mellitus with hyperglycemia |
| E0969 | Drug or chemical induced diabetes mellitus with other specified complication |
| E098 | Drug or chemical induced diabetes mellitus with unspecified complications |
| E1021 | Type 1 diabetes mellitus with diabetic nephropathy |
| E1022 | Type 1 diabetes mellitus with diabetic chronic kidney disease |
| E1029 | Type 1 diabetes mellitus with other diabetic kidney complication |
| E10311 | Type 1 diabetes mellitus with unspecified diabetic retinopathy with macular edema |
| E10319 | Type 1 diabetes mellitus with unspecified diabetic retinopathy without macular edema |
| E10321 | Type 1 diabetes mellitus with mild nonproliferative diabetic retinopathy with macular edema |
| E103211 | Type 1 diabetes mellitus with mild nonproliferative diabetic retinopathy with macular edema, right eye |
| E103212 | Type 1 diabetes mellitus with mild nonproliferative diabetic retinopathy with macular edema, left eye |
| E103213 | Type 1 diabetes mellitus with mild nonproliferative diabetic retinopathy with macular edema, bilateral |
| E103219 | Type 1 diabetes mellitus with mild nonproliferative diabetic retinopathy with macular edema, unspecified eye |
| E10329 | Type 1 diabetes mellitus with mild nonproliferative diabetic retinopathy without macular edema |
| E103291 | Type 1 diabetes mellitus with mild nonproliferative diabetic retinopathy without macular edema, right eye |
| E103292 | Type 1 diabetes mellitus with mild nonproliferative diabetic retinopathy without macular edema, left eye |
| E103293 | Type 1 diabetes mellitus with mild nonproliferative diabetic retinopathy without macular edema, bilateral |
| E103299 | Type 1 diabetes mellitus with mild nonproliferative diabetic retinopathy without macular edema, unspecified eye |
| E10331 | Type 1 diabetes mellitus with moderate nonproliferative diabetic retinopathy with macular edema |
| E103311 | Type 1 diabetes mellitus with moderate nonproliferative diabetic retinopathy with macular edema, right eye |
| E103312 | Type 1 diabetes mellitus with moderate nonproliferative diabetic retinopathy with macular edema, left eye |
| E103313 | Type 1 diabetes mellitus with moderate nonproliferative diabetic retinopathy with macular edema, bilateral |
| E103319 | Type 1 diabetes mellitus with moderate nonproliferative diabetic retinopathy with macular edema, unspecified eye |
| E10339 | Type 1 diabetes mellitus with moderate nonproliferative diabetic retinopathy without macular edema |
| E103391 | Type 1 diabetes mellitus with moderate nonproliferative diabetic retinopathy without macular edema, right eye |
| E103392 | Type 1 diabetes mellitus with moderate nonproliferative diabetic retinopathy without macular edema, left eye |
| E103393 | Type 1 diabetes mellitus with moderate nonproliferative diabetic retinopathy without macular edema, bilateral |
| E103399 | Type 1 diabetes mellitus with moderate nonproliferative diabetic retinopathy without macular edema, unspecified eye |
| E10341 | Type 1 diabetes mellitus with severe nonproliferative diabetic retinopathy with macular edema |
| E103411 | Type 1 diabetes mellitus with severe nonproliferative diabetic retinopathy with macular edema, right eye |
| E103412 | Type 1 diabetes mellitus with severe nonproliferative diabetic retinopathy with macular edema, left eye |
| E103413 | Type 1 diabetes mellitus with severe nonproliferative diabetic retinopathy with macular edema, bilateral |
| E103419 | Type 1 diabetes mellitus with severe nonproliferative diabetic retinopathy with macular edema, unspecified eye |
| E10349 | Type 1 diabetes mellitus with severe nonproliferative diabetic retinopathy without macular edema |
| E103491 | Type 1 diabetes mellitus with severe nonproliferative diabetic retinopathy without macular edema, right eye |
| E103492 | Type 1 diabetes mellitus with severe nonproliferative diabetic retinopathy without macular edema, left eye |
| E103493 | Type 1 diabetes mellitus with severe nonproliferative diabetic retinopathy without macular edema, bilateral |
| E103499 | Type 1 diabetes mellitus with severe nonproliferative diabetic retinopathy without macular edema, unspecified eye |
| E10351 | Type 1 diabetes mellitus with proliferative diabetic retinopathy with macular edema |
| E103511 | Type 1 diabetes mellitus with proliferative diabetic retinopathy with macular edema, right eye |
| E103512 | Type 1 diabetes mellitus with proliferative diabetic retinopathy with macular edema, left eye |
| E103513 | Type 1 diabetes mellitus with proliferative diabetic retinopathy with macular edema, bilateral |
| E103519 | Type 1 diabetes mellitus with proliferative diabetic retinopathy with macular edema, unspecified eye |
| E103521 | Type 1 diabetes mellitus with proliferative diabetic retinopathy with traction retinal detachment involving the macula, right eye |
| E103522 | Type 1 diabetes mellitus with proliferative diabetic retinopathy with traction retinal detachment involving the macula, left eye |
| E103523 | Type 1 diabetes mellitus with proliferative diabetic retinopathy with traction retinal detachment involving the macula, bilateral |
| E103529 | Type 1 diabetes mellitus with proliferative diabetic retinopathy with traction retinal detachment involving the macula, unspecified eye |
| E103531 | Type 1 diabetes mellitus with proliferative diabetic retinopathy with traction retinal detachment not involving the macula, right eye |
| E103532 | Type 1 diabetes mellitus with proliferative diabetic retinopathy with traction retinal detachment not involving the macula, left eye |
| E103533 | Type 1 diabetes mellitus with proliferative diabetic retinopathy with traction retinal detachment not involving the macula, bilateral |
| E103539 | Type 1 diabetes mellitus with proliferative diabetic retinopathy with traction retinal detachment not involving the macula, unspecified eye |
| E103541 | Type 1 diabetes mellitus with proliferative diabetic retinopathy with combined traction retinal detachment and rhegmatogenous retinal detachment, right eye |
| E103542 | Type 1 diabetes mellitus with proliferative diabetic retinopathy with combined traction retinal detachment and rhegmatogenous retinal detachment, left eye |
| E103543 | Type 1 diabetes mellitus with proliferative diabetic retinopathy with combined traction retinal detachment and rhegmatogenous retinal detachment, bilateral |
| E103549 | Type 1 diabetes mellitus with proliferative diabetic retinopathy with combined traction retinal detachment and rhegmatogenous retinal detachment, unspecified eye |
| E103551 | Type 1 diabetes mellitus with stable proliferative diabetic retinopathy, right eye |
| E103552 | Type 1 diabetes mellitus with stable proliferative diabetic retinopathy, left eye |
| E103553 | Type 1 diabetes mellitus with stable proliferative diabetic retinopathy, bilateral |
| E103559 | Type 1 diabetes mellitus with stable proliferative diabetic retinopathy, unspecified eye |
| E10359 | Type 1 diabetes mellitus with proliferative diabetic retinopathy without macular edema |
| E103591 | Type 1 diabetes mellitus with proliferative diabetic retinopathy without macular edema, right eye |
| E103592 | Type 1 diabetes mellitus with proliferative diabetic retinopathy without macular edema, left eye |
| E103593 | Type 1 diabetes mellitus with proliferative diabetic retinopathy without macular edema, bilateral |
| E103599 | Type 1 diabetes mellitus with proliferative diabetic retinopathy without macular edema, unspecified eye |
| E1036 | Type 1 diabetes mellitus with diabetic cataract |
| E1037X1 | Type 1 diabetes mellitus with diabetic macular edema, resolved following treatment, right eye |
| E1037X2 | Type 1 diabetes mellitus with diabetic macular edema, resolved following treatment, left eye |
| E1037X3 | Type 1 diabetes mellitus with diabetic macular edema, resolved following treatment, bilateral |
| E1037X9 | Type 1 diabetes mellitus with diabetic macular edema, resolved following treatment, unspecified eye |
| E1039 | Type 1 diabetes mellitus with other diabetic ophthalmic complication |
| E1040 | Type 1 diabetes mellitus with diabetic neuropathy, unspecified |
| E1041 | Type 1 diabetes mellitus with diabetic mononeuropathy |
| E1042 | Type 1 diabetes mellitus with diabetic polyneuropathy |
| E1043 | Type 1 diabetes mellitus with diabetic autonomic (poly)neuropathy |
| E1044 | Type 1 diabetes mellitus with diabetic amyotrophy |
| E1049 | Type 1 diabetes mellitus with other diabetic neurological complication |
| E1051 | Type 1 diabetes mellitus with diabetic peripheral angiopathy without gangrene |
| E1052 | Type 1 diabetes mellitus with diabetic peripheral angiopathy with gangrene |
| E1059 | Type 1 diabetes mellitus with other circulatory complications |
| E10610 | Type 1 diabetes mellitus with diabetic neuropathic arthropathy |
| E10618 | Type 1 diabetes mellitus with other diabetic arthropathy |
| E10620 | Type 1 diabetes mellitus with diabetic dermatitis |
| E10621 | Type 1 diabetes mellitus with foot ulcer |
| E10622 | Type 1 diabetes mellitus with other skin ulcer |
| E10628 | Type 1 diabetes mellitus with other skin complications |
| E10630 | Type 1 diabetes mellitus with periodontal disease |
| E10638 | Type 1 diabetes mellitus with other oral complications |
| E10641 | Type 1 diabetes mellitus with hypoglycemia with coma |
| E10649 | Type 1 diabetes mellitus with hypoglycemia without coma |
| E1065 | Type 1 diabetes mellitus with hyperglycemia |
| E1069 | Type 1 diabetes mellitus with other specified complication |
| E108 | Type 1 diabetes mellitus with unspecified complications |
| E1121 | Type 2 diabetes mellitus with diabetic nephropathy |
| E1122 | Type 2 diabetes mellitus with diabetic chronic kidney disease |
| E1129 | Type 2 diabetes mellitus with other diabetic kidney complication |
| E11311 | Type 2 diabetes mellitus with unspecified diabetic retinopathy with macular edema |
| E11319 | Type 2 diabetes mellitus with unspecified diabetic retinopathy without macular edema |
| E11321 | Type 2 diabetes mellitus with mild nonproliferative diabetic retinopathy with macular edema |
| E113211 | Type 2 diabetes mellitus with mild nonproliferative diabetic retinopathy with macular edema, right eye |
| E113212 | Type 2 diabetes mellitus with mild nonproliferative diabetic retinopathy with macular edema, left eye |
| E113213 | Type 2 diabetes mellitus with mild nonproliferative diabetic retinopathy with macular edema, bilateral |
| E113219 | Type 2 diabetes mellitus with mild nonproliferative diabetic retinopathy with macular edema, unspecified eye |
| E11329 | Type 2 diabetes mellitus with mild nonproliferative diabetic retinopathy without macular edema |
| E113291 | Type 2 diabetes mellitus with mild nonproliferative diabetic retinopathy without macular edema, right eye |
| E113292 | Type 2 diabetes mellitus with mild nonproliferative diabetic retinopathy without macular edema, left eye |
| E113293 | Type 2 diabetes mellitus with mild nonproliferative diabetic retinopathy without macular edema, bilateral |
| E113299 | Type 2 diabetes mellitus with mild nonproliferative diabetic retinopathy without macular edema, unspecified eye |
| E11331 | Type 2 diabetes mellitus with moderate nonproliferative diabetic retinopathy with macular edema |
| E113311 | Type 2 diabetes mellitus with moderate nonproliferative diabetic retinopathy with macular edema, right eye |
| E113312 | Type 2 diabetes mellitus with moderate nonproliferative diabetic retinopathy with macular edema, left eye |
| E113313 | Type 2 diabetes mellitus with moderate nonproliferative diabetic retinopathy with macular edema, bilateral |
| E113319 | Type 2 diabetes mellitus with moderate nonproliferative diabetic retinopathy with macular edema, unspecified eye |
| E11339 | Type 2 diabetes mellitus with moderate nonproliferative diabetic retinopathy without macular edema |
| E113391 | Type 2 diabetes mellitus with moderate nonproliferative diabetic retinopathy without macular edema, right eye |
| E113392 | Type 2 diabetes mellitus with moderate nonproliferative diabetic retinopathy without macular edema, left eye |
| E113393 | Type 2 diabetes mellitus with moderate nonproliferative diabetic retinopathy without macular edema, bilateral |
| E113399 | Type 2 diabetes mellitus with moderate nonproliferative diabetic retinopathy without macular edema, unspecified eye |
| E11341 | Type 2 diabetes mellitus with severe nonproliferative diabetic retinopathy with macular edema |
| E113411 | Type 2 diabetes mellitus with severe nonproliferative diabetic retinopathy with macular edema, right eye |
| E113412 | Type 2 diabetes mellitus with severe nonproliferative diabetic retinopathy with macular edema, left eye |
| E113413 | Type 2 diabetes mellitus with severe nonproliferative diabetic retinopathy with macular edema, bilateral |
| E113419 | Type 2 diabetes mellitus with severe nonproliferative diabetic retinopathy with macular edema, unspecified eye |
| E11349 | Type 2 diabetes mellitus with severe nonproliferative diabetic retinopathy without macular edema |
| E113491 | Type 2 diabetes mellitus with severe nonproliferative diabetic retinopathy without macular edema, right eye |
| E113492 | Type 2 diabetes mellitus with severe nonproliferative diabetic retinopathy without macular edema, left eye |
| E113493 | Type 2 diabetes mellitus with severe nonproliferative diabetic retinopathy without macular edema, bilateral |
| E113499 | Type 2 diabetes mellitus with severe nonproliferative diabetic retinopathy without macular edema, unspecified eye |
| E11351 | Type 2 diabetes mellitus with proliferative diabetic retinopathy with macular edema |
| E113511 | Type 2 diabetes mellitus with proliferative diabetic retinopathy with macular edema, right eye |
| E113512 | Type 2 diabetes mellitus with proliferative diabetic retinopathy with macular edema, left eye |
| E113513 | Type 2 diabetes mellitus with proliferative diabetic retinopathy with macular edema, bilateral |
| E113519 | Type 2 diabetes mellitus with proliferative diabetic retinopathy with macular edema, unspecified eye |
| E113521 | Type 2 diabetes mellitus with proliferative diabetic retinopathy with traction retinal detachment involving the macula, right eye |
| E113522 | Type 2 diabetes mellitus with proliferative diabetic retinopathy with traction retinal detachment involving the macula, left eye |
| E113523 | Type 2 diabetes mellitus with proliferative diabetic retinopathy with traction retinal detachment involving the macula, bilateral |
| E113529 | Type 2 diabetes mellitus with proliferative diabetic retinopathy with traction retinal detachment involving the macula, unspecified eye |
| E113531 | Type 2 diabetes mellitus with proliferative diabetic retinopathy with traction retinal detachment not involving the macula, right eye |
| E113532 | Type 2 diabetes mellitus with proliferative diabetic retinopathy with traction retinal detachment not involving the macula, left eye |
| E113533 | Type 2 diabetes mellitus with proliferative diabetic retinopathy with traction retinal detachment not involving the macula, bilateral |
| E113539 | Type 2 diabetes mellitus with proliferative diabetic retinopathy with traction retinal detachment not involving the macula, unspecified eye |
| E113541 | Type 2 diabetes mellitus with proliferative diabetic retinopathy with combined traction retinal detachment and rhegmatogenous retinal detachment, right eye |
| E113542 | Type 2 diabetes mellitus with proliferative diabetic retinopathy with combined traction retinal detachment and rhegmatogenous retinal detachment, left eye |
| E113543 | Type 2 diabetes mellitus with proliferative diabetic retinopathy with combined traction retinal detachment and rhegmatogenous retinal detachment, bilateral |
| E113549 | Type 2 diabetes mellitus with proliferative diabetic retinopathy with combined traction retinal detachment and rhegmatogenous retinal detachment, unspecified eye |
| E113551 | Type 2 diabetes mellitus with stable proliferative diabetic retinopathy, right eye |
| E113552 | Type 2 diabetes mellitus with stable proliferative diabetic retinopathy, left eye |
| E113553 | Type 2 diabetes mellitus with stable proliferative diabetic retinopathy, bilateral |
| E113559 | Type 2 diabetes mellitus with stable proliferative diabetic retinopathy, unspecified eye |
| E11359 | Type 2 diabetes mellitus with proliferative diabetic retinopathy without macular edema |
| E113591 | Type 2 diabetes mellitus with proliferative diabetic retinopathy without macular edema, right eye |
| E113592 | Type 2 diabetes mellitus with proliferative diabetic retinopathy without macular edema, left eye |
| E113593 | Type 2 diabetes mellitus with proliferative diabetic retinopathy without macular edema, bilateral |
| E113599 | Type 2 diabetes mellitus with proliferative diabetic retinopathy without macular edema, unspecified eye |
| E1136 | Type 2 diabetes mellitus with diabetic cataract |
| E1137X1 | Type 2 diabetes mellitus with diabetic macular edema, resolved following treatment, right eye |
| E1137X2 | Type 2 diabetes mellitus with diabetic macular edema, resolved following treatment, left eye |
| E1137X3 | Type 2 diabetes mellitus with diabetic macular edema, resolved following treatment, bilateral |
| E1137X9 | Type 2 diabetes mellitus with diabetic macular edema, resolved following treatment, unspecified eye |
| E1139 | Type 2 diabetes mellitus with other diabetic ophthalmic complication |
| E1140 | Type 2 diabetes mellitus with diabetic neuropathy, unspecified |
| E1141 | Type 2 diabetes mellitus with diabetic mononeuropathy |
| E1142 | Type 2 diabetes mellitus with diabetic polyneuropathy |
| E1143 | Type 2 diabetes mellitus with diabetic autonomic (poly)neuropathy |
| E1144 | Type 2 diabetes mellitus with diabetic amyotrophy |
| E1149 | Type 2 diabetes mellitus with other diabetic neurological complication |
| E1151 | Type 2 diabetes mellitus with diabetic peripheral angiopathy without gangrene |
| E1152 | Type 2 diabetes mellitus with diabetic peripheral angiopathy with gangrene |
| E1159 | Type 2 diabetes mellitus with other circulatory complications |
| E11610 | Type 2 diabetes mellitus with diabetic neuropathic arthropathy |
| E11618 | Type 2 diabetes mellitus with other diabetic arthropathy |
| E11620 | Type 2 diabetes mellitus with diabetic dermatitis |
| E11621 | Type 2 diabetes mellitus with foot ulcer |
| E11622 | Type 2 diabetes mellitus with other skin ulcer |
| E11628 | Type 2 diabetes mellitus with other skin complications |
| E11630 | Type 2 diabetes mellitus with periodontal disease |
| E11638 | Type 2 diabetes mellitus with other oral complications |
| E11641 | Type 2 diabetes mellitus with hypoglycemia with coma |
| E11649 | Type 2 diabetes mellitus with hypoglycemia without coma |
| E1165 | Type 2 diabetes mellitus with hyperglycemia |
| E1169 | Type 2 diabetes mellitus with other specified complication |
| E118 | Type 2 diabetes mellitus with unspecified complications |
| E1321 | Other specified diabetes mellitus with diabetic nephropathy |
| E1322 | Other specified diabetes mellitus with diabetic chronic kidney disease |
| E1329 | Other specified diabetes mellitus with other diabetic kidney complication |
| E13311 | Other specified diabetes mellitus with unspecified diabetic retinopathy with macular edema |
| E13319 | Other specified diabetes mellitus with unspecified diabetic retinopathy without macular edema |
| E13321 | Other specified diabetes mellitus with mild nonproliferative diabetic retinopathy with macular edema |
| E133211 | Other specified diabetes mellitus with mild nonproliferative diabetic retinopathy with macular edema, right eye |
| E133212 | Other specified diabetes mellitus with mild nonproliferative diabetic retinopathy with macular edema, left eye |
| E133213 | Other specified diabetes mellitus with mild nonproliferative diabetic retinopathy with macular edema, bilateral |
| E133219 | Other specified diabetes mellitus with mild nonproliferative diabetic retinopathy with macular edema, unspecified eye |
| E13329 | Other specified diabetes mellitus with mild nonproliferative diabetic retinopathy without macular edema |
| E133291 | Other specified diabetes mellitus with mild nonproliferative diabetic retinopathy without macular edema, right eye |
| E133292 | Other specified diabetes mellitus with mild nonproliferative diabetic retinopathy without macular edema, left eye |
| E133293 | Other specified diabetes mellitus with mild nonproliferative diabetic retinopathy without macular edema, bilateral |
| E133299 | Other specified diabetes mellitus with mild nonproliferative diabetic retinopathy without macular edema, unspecified eye |
| E13331 | Other specified diabetes mellitus with moderate nonproliferative diabetic retinopathy with macular edema |
| E133311 | Other specified diabetes mellitus with moderate nonproliferative diabetic retinopathy with macular edema, right eye |
| E133312 | Other specified diabetes mellitus with moderate nonproliferative diabetic retinopathy with macular edema, left eye |
| E133313 | Other specified diabetes mellitus with moderate nonproliferative diabetic retinopathy with macular edema, bilateral |
| E133319 | Other specified diabetes mellitus with moderate nonproliferative diabetic retinopathy with macular edema, unspecified eye |
| E13339 | Other specified diabetes mellitus with moderate nonproliferative diabetic retinopathy without macular edema |
| E133391 | Other specified diabetes mellitus with moderate nonproliferative diabetic retinopathy without macular edema, right eye |
| E133392 | Other specified diabetes mellitus with moderate nonproliferative diabetic retinopathy without macular edema, left eye |
| E133393 | Other specified diabetes mellitus with moderate nonproliferative diabetic retinopathy without macular edema, bilateral |
| E133399 | Other specified diabetes mellitus with moderate nonproliferative diabetic retinopathy without macular edema, unspecified eye |
| E13341 | Other specified diabetes mellitus with severe nonproliferative diabetic retinopathy with macular edema |
| E133411 | Other specified diabetes mellitus with severe nonproliferative diabetic retinopathy with macular edema, right eye |
| E133412 | Other specified diabetes mellitus with severe nonproliferative diabetic retinopathy with macular edema, left eye |
| E133413 | Other specified diabetes mellitus with severe nonproliferative diabetic retinopathy with macular edema, bilateral |
| E133419 | Other specified diabetes mellitus with severe nonproliferative diabetic retinopathy with macular edema, unspecified eye |
| E13349 | Other specified diabetes mellitus with severe nonproliferative diabetic retinopathy without macular edema |
| E133491 | Other specified diabetes mellitus with severe nonproliferative diabetic retinopathy without macular edema, right eye |
| E133492 | Other specified diabetes mellitus with severe nonproliferative diabetic retinopathy without macular edema, left eye |
| E133493 | Other specified diabetes mellitus with severe nonproliferative diabetic retinopathy without macular edema, bilateral |
| E133499 | Other specified diabetes mellitus with severe nonproliferative diabetic retinopathy without macular edema, unspecified eye |
| E13351 | Other specified diabetes mellitus with proliferative diabetic retinopathy with macular edema |
| E133511 | Other specified diabetes mellitus with proliferative diabetic retinopathy with macular edema, right eye |
| E133512 | Other specified diabetes mellitus with proliferative diabetic retinopathy with macular edema, left eye |
| E133513 | Other specified diabetes mellitus with proliferative diabetic retinopathy with macular edema, bilateral |
| E133519 | Other specified diabetes mellitus with proliferative diabetic retinopathy with macular edema, unspecified eye |
| E133521 | Other specified diabetes mellitus with proliferative diabetic retinopathy with traction retinal detachment involving the macula, right eye |
| E133522 | Other specified diabetes mellitus with proliferative diabetic retinopathy with traction retinal detachment involving the macula, left eye |
| E133523 | Other specified diabetes mellitus with proliferative diabetic retinopathy with traction retinal detachment involving the macula, bilateral |
| E133529 | Other specified diabetes mellitus with proliferative diabetic retinopathy with traction retinal detachment involving the macula, unspecified eye |
| E133531 | Other specified diabetes mellitus with proliferative diabetic retinopathy with traction retinal detachment not involving the macula, right eye |
| E133532 | Other specified diabetes mellitus with proliferative diabetic retinopathy with traction retinal detachment not involving the macula, left eye |
| E133533 | Other specified diabetes mellitus with proliferative diabetic retinopathy with traction retinal detachment not involving the macula, bilateral |
| E133539 | Other specified diabetes mellitus with proliferative diabetic retinopathy with traction retinal detachment not involving the macula, unspecified eye |
| E133541 | Other specified diabetes mellitus with proliferative diabetic retinopathy with combined traction retinal detachment and rhegmatogenous retinal detachment, right eye |
| E133542 | Other specified diabetes mellitus with proliferative diabetic retinopathy with combined traction retinal detachment and rhegmatogenous retinal detachment, left eye |
| E133543 | Other specified diabetes mellitus with proliferative diabetic retinopathy with combined traction retinal detachment and rhegmatogenous retinal detachment, bilateral |
| E133549 | Other specified diabetes mellitus with proliferative diabetic retinopathy with combined traction retinal detachment and rhegmatogenous retinal detachment, unspecified eye |
| E133551 | Other specified diabetes mellitus with stable proliferative diabetic retinopathy, right eye |
| E133552 | Other specified diabetes mellitus with stable proliferative diabetic retinopathy, left eye |
| E133553 | Other specified diabetes mellitus with stable proliferative diabetic retinopathy, bilateral |
| E133559 | Other specified diabetes mellitus with stable proliferative diabetic retinopathy, unspecified eye |
| E13359 | Other specified diabetes mellitus with proliferative diabetic retinopathy without macular edema |
| E133591 | Other specified diabetes mellitus with proliferative diabetic retinopathy without macular edema, right eye |
| E133592 | Other specified diabetes mellitus with proliferative diabetic retinopathy without macular edema, left eye |
| E133593 | Other specified diabetes mellitus with proliferative diabetic retinopathy without macular edema, bilateral |
| E133599 | Other specified diabetes mellitus with proliferative diabetic retinopathy without macular edema, unspecified eye |
| E1336 | Other specified diabetes mellitus with diabetic cataract |
| E1337X1 | Other specified diabetes mellitus with diabetic macular edema, resolved following treatment, right eye |
| E1337X2 | Other specified diabetes mellitus with diabetic macular edema, resolved following treatment, left eye |
| E1337X3 | Other specified diabetes mellitus with diabetic macular edema, resolved following treatment, bilateral |
| E1337X9 | Other specified diabetes mellitus with diabetic macular edema, resolved following treatment, unspecified eye |
| E1339 | Other specified diabetes mellitus with other diabetic ophthalmic complication |
| E1340 | Other specified diabetes mellitus with diabetic neuropathy, unspecified |
| E1341 | Other specified diabetes mellitus with diabetic mononeuropathy |
| E1342 | Other specified diabetes mellitus with diabetic polyneuropathy |
| E1343 | Other specified diabetes mellitus with diabetic autonomic (poly)neuropathy |
| E1344 | Other specified diabetes mellitus with diabetic amyotrophy |
| E1349 | Other specified diabetes mellitus with other diabetic neurological complication |
| E1351 | Other specified diabetes mellitus with diabetic peripheral angiopathy without gangrene |
| E1352 | Other specified diabetes mellitus with diabetic peripheral angiopathy with gangrene |
| E1359 | Other specified diabetes mellitus with other circulatory complications |
| E13610 | Other specified diabetes mellitus with diabetic neuropathic arthropathy |
| E13618 | Other specified diabetes mellitus with other diabetic arthropathy |
| E13620 | Other specified diabetes mellitus with diabetic dermatitis |
| E13621 | Other specified diabetes mellitus with foot ulcer |
| E13622 | Other specified diabetes mellitus with other skin ulcer |
| E13628 | Other specified diabetes mellitus with other skin complications |
| E13630 | Other specified diabetes mellitus with periodontal disease |
| E13638 | Other specified diabetes mellitus with other oral complications |
| E13641 | Other specified diabetes mellitus with hypoglycemia with coma |
| E13649 | Other specified diabetes mellitus with hypoglycemia without coma |
| E1365 | Other specified diabetes mellitus with hyperglycemia |
| E1369 | Other specified diabetes mellitus with other specified complication |
| E138 | Other specified diabetes mellitus with unspecified complications |

## Table S3. ICD- 10 Codes Used to Define Heart Failure (n = 37)

| **ICD- 10 Codes Used to Define Heart Failure** (n = 37) | |
| --- | --- |
| I0981 | Rheumatic heart failure |
| I110 | Hypertensive heart disease with heart failure |
| I130 | Hypertensive heart and chronic kidney disease with heart failure and stage 1 through stage 4 chronic kidney disease, or unspecified chronic kidney disease |
| I132 | Hypertensive heart and chronic kidney disease with heart failure and with stage 5 chronic kidney disease, or end stage renal disease |
| I501 | Left ventricular failure, unspecified |
| I5020 | Unspecified systolic (congestive) heart failure |
| I5021 | Acute systolic (congestive) heart failure |
| I5022 | Chronic systolic (congestive) heart failure |
| I5023 | Acute on chronic systolic (congestive) heart failure |
| I5030 | Unspecified diastolic (congestive) heart failure |
| I5031 | Acute diastolic (congestive) heart failure |
| I5032 | Chronic diastolic (congestive) heart failure |
| I5033 | Acute on chronic diastolic (congestive) heart failure |
| I5040 | Unspecified combined systolic (congestive) and diastolic (congestive) heart failure |
| I5041 | Acute combined systolic (congestive) and diastolic (congestive) heart failure |
| I5042 | Chronic combined systolic (congestive) and diastolic (congestive) heart failure |
| I5043 | Acute on chronic combined systolic (congestive) and diastolic (congestive) heart failure |
| I50810 | Right heart failure, unspecified |
| I50811 | Acute right heart failure |
| I50812 | Chronic right heart failure |
| I50813 | Acute on chronic right heart failure |
| I50814 | Right heart failure due to left heart failure |
| I5082 | Biventricular heart failure |
| I5083 | High output heart failure |
| I5084 | End stage heart failure |
| I5089 | Other heart failure |
| I509 | Heart failure, unspecified |
| I5181 | Takotsubo syndrome |
| I97130 | Postprocedural heart failure following cardiac surgery |
| I97131 | Postprocedural heart failure following other surgery |
| O29121 | Cardiac failure due to anesthesia during pregnancy, first trimester |
| O29122 | Cardiac failure due to anesthesia during pregnancy, second trimester |
| O29123 | Cardiac failure due to anesthesia during pregnancy, third trimester |
| O29129 | Cardiac failure due to anesthesia during pregnancy, unspecified trimester |
| R570 | Cardiogenic shock |
| Z95811 | Presence of heart assist device |
| Z95812 | Presence of fully implantable artificial heart |

## Table S4. ICD- 10 Codes Used to Define Obesity (n = 36)

| **ICD- 10 Codes Used to Define Obesity** (n = 36) | |
| --- | --- |
| E6601 | Morbid (severe) obesity due to excess calories |
| E6609 | Other obesity due to excess calories |
| E661 | Drug-induced obesity |
| E662 | Morbid (severe) obesity with alveolar hypoventilation |
| E668 | Other obesity |
| E66811 | Obesity, class 1 |
| E66812 | Obesity, class 2 |
| E66813 | Obesity, class 3 |
| E6689 | Other obesity not elsewhere classified |
| E669 | Obesity, unspecified |
| E8882 | Obesity due to disruption of MC4R pathway |
| O99210 | Obesity complicating pregnancy, unspecified trimester |
| O99211 | Obesity complicating pregnancy, first trimester |
| O99212 | Obesity complicating pregnancy, second trimester |
| O99213 | Obesity complicating pregnancy, third trimester |
| O99214 | Obesity complicating childbirth |
| O99215 | Obesity complicating the puerperium |
| R939 | Diagnostic imaging inconclusive due to excess body fat of patient |
| Z6830 | Body mass index [BMI] 30.0-30.9, adult |
| Z6831 | Body mass index [BMI] 31.0-31.9, adult |
| Z6832 | Body mass index [BMI] 32.0-32.9, adult |
| Z6833 | Body mass index [BMI] 33.0-33.9, adult |
| Z6834 | Body mass index [BMI] 34.0-34.9, adult |
| Z6835 | Body mass index [BMI] 35.0-35.9, adult |
| Z6836 | Body mass index [BMI] 36.0-36.9, adult |
| Z6837 | Body mass index [BMI] 37.0-37.9, adult |
| Z6838 | Body mass index [BMI] 38.0-38.9, adult |
| Z6839 | Body mass index [BMI] 39.0-39.9, adult |
| Z6841 | Body mass index [BMI] 40.0-44.9, adult |
| Z6842 | Body mass index [BMI] 45.0-49.9, adult |
| Z6843 | Body mass index [BMI] 50.0-59.9, adult |
| Z6844 | Body mass index [BMI] 60.0-69.9, adult |
| Z6845 | Body mass index [BMI] 70 or greater, adult |
| Z6854 | Body mass index [BMI] pediatric, greater than or equal to 95th percentile for age |
| Z6855 | Body mass index [BMI] pediatric, 120% of the 95th percentile for age to less than 140% of the 95th percentile for age |
| Z6856 | Body mass index [BMI] pediatric, greater than or equal to 140% of the 95th percentile for age |

## Table S5. ICD- 10 Codes Used to Define Weight Loss (n = 18)

| **ICD- 10 Codes Used to Define Weight Loss** (n = 18) | |
| --- | --- |
| E40 | Kwashiorkor |
| E41 | Nutritional marasmus |
| E42 | Marasmic kwashiorkor |
| E43 | Unspecified severe protein-calorie malnutrition |
| E440 | Moderate protein-calorie malnutrition |
| E441 | Mild protein-calorie malnutrition |
| E45 | Retarded development following protein-calorie malnutrition |
| E46 | Unspecified protein-calorie malnutrition |
| E640 | Sequelae of protein-calorie malnutrition |
| E88A | Wasting disease (syndrome) due to underlying condition |
| O2510 | Malnutrition in pregnancy, unspecified trimester |
| O2511 | Malnutrition in pregnancy, first trimester |
| O2512 | Malnutrition in pregnancy, second trimester |
| O2513 | Malnutrition in pregnancy, third trimester |
| O252 | Malnutrition in childbirth |
| O253 | Malnutrition in the puerperium |
| R634 | Abnormal weight loss |
| R64 | Cachexia |

## Table S6. NTA Point Values Associated with Documentation-Sensitive Conditions

| **Documentation-Sensitive Condition** | **NTA-Relevant Conditions** | **NTA Points** | **Percent of codes present on NTA list** |
| --- | --- | --- | --- |
| Chronic Pulmonary Disease | Pulmonary Fibrosis and Other Chronic Lung Disorders  Respiratory Arrest | 1 | 7 % |
| Complicated Diabetes | Diabetes Mellitus (DM) Code | 2 | 56% |
| Heart Failure | Cardio-Respiratory Failure and Shock  Endocarditis | 1 | 3% |
| Obesity | Morbid Obesity | 1 | 19% |
| Weight loss | NA | NA | 0% |

Table S6: Documentation-sensitive conditions, their closest corresponding PDPM Non-Therapy Ancillary (NTA) comorbidity category and point value, and the proportion of diagnosis codes used to define each documentation-sensitive condition that also appear on the official NTA comorbidity list.

## Table S7. Full Regression Results: Diagnosis Count among all SNFs

| Full Regression Results: Diagnosis Count among all SNFs (n= 9,335,052) | | | | | |
| --- | --- | --- | --- | --- | --- |
| **Term** | **Estimate** | **Std. Error** | **t value** | **Pr(>\|t\|)** | **Stars** |
| Hospital Value-Based Purchasing Participant | 1.057138 | 0.0483 | 21.88499 | < 2.2e-16 | *** |
| Hospital ACO Participant | 0.524558 | 0.0742 | 7.068456 | 9.07E-09 | *** |
| For-profit Hospital | -0.25121 | 0.0229 | -10.9587 | 3.68E-14 | *** |
| Hospital with Affiliated SNFs | 0.045042 | 0.030179 | 1.492499 | 1.43E-01 |  |
| For-profit SNF | 0.339575 | 0.0184 | 18.44009 | < 2.2e-16 | *** |
| Chain SNF | 0.110094 | 0.0163 | 6.740838 | 2.74E-08 | *** |
| Medicare SNF Bed Count | -0.00307 | 0.000357 | -8.59234 | 5.78E-11 | *** |
| Overall 5-star NH rating | -0.14393 | 0.00824 | -17.471 | < 2.2e-16 | *** |
| Hospital Rurality: large rural | -0.55859 | 0.075 | -7.44881 | 2.53E-09 | *** |
| Hospital Rurality: small rural | -0.2072 | 0.0714 | -2.90315 | 5.76E-03 | ** |
| Hospital Rurality: urban | -0.52043 | 0.0778 | -6.68918 | 3.27E-08 | *** |
| SNF Rurality: large rural | 0.05304 | 0.0316 | 1.677038 | 1.01E-01 |  |
| SNF Rurality: small rural | -0.02188 | 0.0315 | -0.6955 | 4.90E-01 |  |
| SNF Rurality: urban | 0.011025 | 0.0322 | 0.342332 | 7.34E-01 |  |
| Local per capita facility-level COVID-19 | 1.200747 | 1.15 | 1.046761 | 3.01E-01 |  |
| Treatment*Post PDPM Announcement | 0.827658 | 0.104702 | 7.904879 | 5.53E-10 | *** |
| Significance. codes: 0 ‘***’ 0.001 ‘**’ 0.01 ‘*’ 0.05 ‘.’ 0.1 ‘ ’ 1 | | | | | |

## Table S8. Full Regression Results: Diagnosis Count with Profit-Status interaction, among all SNFs

| Full Regression Results: Diagnosis Count with SNF-profit interaction among all SNFs (n= 9,335,052) | | | | | |
| --- | --- | --- | --- | --- | --- |
| **Term** | **Estimate** | **Std. Error** | **t value** | **Pr(>\|t\|)** | **Stars** |
| For-Profit SNF | 0.391985 | 0.029118 | 13.46217 | < 2.2e-16 | *** |
| Hospital Value-Based Purchasing Participant | 1.057152 | 0.048305 | 21.88507 | < 2.2e-16 | *** |
| Hospital ACO Participant | 0.523827 | 0.074225 | 7.057328 | 0.00 | *** |
| For-profit hospital | -0.25105 | 0.022942 | -10.9425 | 0.00 | *** |
| Hospital with Affiliated SNFs | 0.045005 | 0.030194 | 1.490501 | 0.14 |  |
| Chain SNF | 0.108549 | 0.016188 | 6.705675 | 0.00 | *** |
| Medicare SNF Bed Count | -0.00306 | 0.000357 | -8.56127 | 0.00 | *** |
| Overall 5-star NH rating | -0.14306 | 0.008182 | -17.4857 | < 2.2e-16 | *** |
| Hospital Rurality: large rural | -0.55818 | 0.075045 | -7.438 | 0.00 | *** |
| Hospital Rurality: small rural | -0.20698 | 0.071413 | -2.89839 | 0.01 | ** |
| Hospital Rurality: urban | -0.51998 | 0.077866 | -6.67793 | 0.00 | *** |
| SNF Rurality: large rural | 0.052699 | 0.03166 | 1.66456 | 0.10 | . |
| SNF Rurality: small rural | -0.02283 | 0.031504 | -0.72456 | 0.47 |  |
| SNF Rurality: urban | 0.010646 | 0.032234 | 0.330269 | 0.74 |  |
| Local per capita facility-level COVID-19 | 1.209406 | 1.147784 | 1.053687 | 0.30 |  |
| Treatment*Post PDPM Announcement | 0.719439 | 0.100159 | 7.182963 | 0.00 | *** |
| Treatment* SNF Profit Status | -0.27461 | 0.146314 | -1.87683 | 0.07 | . |
| Post PDPM Announcement* SNF Profit Status | -0.0512 | 0.033001 | -1.55148 | 0.13 |  |
| Treatment*Post PDPM Announcement*SNF Profit Status | 0.146737 | 0.083408 | 1.759264 | 0.09 | . |
| Significance. codes: 0 ‘***’ 0.001 ‘**’ 0.01 ‘*’ 0.05 ‘.’ 0.1 ‘ ’ 1 | | | | | |

## Table S9. Full Regression Results: Diagnosis Count, among For-Profit SNFs

| Full Regression Results: Diagnosis Count, among all SNFs (n= 6,801,105) | | | | | |
| --- | --- | --- | --- | --- | --- |
| **Term** | **Estimate** | **Std. Error** | **t value** | **Pr(>\|t\|)** | **Stars** |
| Hospital Value-Based Purchasing Participant | 1.132945 | 0.052212 | 21.6991 | < 2.2e-16 | *** |
| Hospital ACO Participant | 0.447398 | 0.082816 | 5.402297 | 2.53E-06 | *** |
| For-profit Hospital | -0.24246 | 0.024321 | -9.9691 | 7.35E-13 | *** |
| Hospital with Affiliated SNFs | 0.018847 | 0.033425 | 0.56387 | 5.76E-01 |  |
| Chain SNF | 0.093796 | 0.019296 | 4.860834 | 1.52E-05 | *** |
| Medicare SNF Bed Count | -0.00349 | 0.000382 | -9.13864 | 9.96E-12 | *** |
| Overall 5-star NH rating | -0.12659 | 0.008751 | -14.5337 | < 2.2e-16 | *** |
| Hospital Rurality: large rural | -0.55217 | 0.091075 | -6.06275 | 2.73E-07 | *** |
| Hospital Rurality: small rural | -0.21726 | 0.086305 | -2.5173 | 1.55E-02 | * |
| Hospital Rurality: urban | -0.51786 | 0.091542 | -5.65702 | 1.07E-06 | *** |
| SNF Rurality: large rural | 0.084032 | 0.039854 | 2.108479 | 4.07E-02 | * |
| SNF Rurality: small rural | -0.00292 | 0.041575 | -0.07023 | 9.44E-01 |  |
| SNF Rurality: urban | 0.067633 | 0.041259 | 1.639214 | 1.08E-01 |  |
| Local per capita facility-level COVID-19 | 1.315755 | 1.265271 | 1.0399 | 3.04E-01 |  |
| Treatment*Post PDPM Announcement | 0.862643 | 0.116148 | 7.427125 | 2.72E-09 | *** |
| Significance. codes: 0 ‘***’ 0.001 ‘**’ 0.01 ‘*’ 0.05 ‘.’ 0.1 ‘ ’ 1 | | | | | |

## Table S10. Full Regression Results: Diagnosis Count, among Not-for-Profit SNFs

| Full Regression Results: Diagnosis Count, among all SNFs (n= 2,533,947) | | | | | |
| --- | --- | --- | --- | --- | --- |
| **Term** | **Estimate** | **Std. Error** | **t value** | **Pr(>\|t\|)** | **Stars** |
| Hospital Value-Based Purchasing Participant | 0.752527 | 0.055177 | 13.63844 | < 2.2e-16 | *** |
| Hospital ACO Participant | 0.647015 | 0.110391 | 5.861121 | 5.40E-07 | *** |
| For-profit Hospital | -0.28356 | 0.038578 | -7.35032 | 3.52E-09 | *** |
| Hospital with Affiliated SNFs | 0.102366 | 0.048314 | 2.118761 | 3.98E-02 | * |
| Chain SNF | 0.11799 | 0.0324 | 3.641726 | 7.10E-04 | *** |
| Medicare SNF Bed Count | -0.00246 | 0.000686 | -3.57847 | 8.56E-04 | *** |
| Overall 5-star NH rating | -0.17074 | 0.014412 | -11.8467 | 2.79E-15 | *** |
| Hospital Rurality: large rural | -0.46648 | 0.124937 | -3.73373 | 5.39E-04 | *** |
| Hospital Rurality: small rural | -0.17549 | 0.125931 | -1.39354 | 1.70E-01 |  |
| Hospital Rurality: urban | -0.40893 | 0.13034 | -3.13741 | 3.04E-03 | ** |
| SNF Rurality: large rural | 0.023976 | 0.06236 | 0.384481 | 7.02E-01 |  |
| SNF Rurality: small rural | -0.06587 | 0.066994 | -0.98321 | 3.31E-01 |  |
| SNF Rurality: urban | -0.0654 | 0.066938 | -0.97699 | 3.34E-01 |  |
| Local per capita facility-level COVID-19 | 0.617887 | 1.696627 | 0.364185 | 7.17E-01 |  |
| Treatment*Post PDPM Announcement | 0.705412 | 0.10095 | 6.987743 | 1.19E-08 | *** |
| Significance. codes: 0 ‘***’ 0.001 ‘**’ 0.01 ‘*’ 0.05 ‘.’ 0.1 ‘ ’ 1 | | | | | |

## Table S11. Full Regression Results: Elixhauser, among all SNFs

| Full Regression Results: Elixhauser, among all SNFs (n= 9,335,052) | | | | | |
| --- | --- | --- | --- | --- | --- |
| **Term** | **Estimate** | **Std. Error** | **t value** | **Pr(>\|t\|)** | **Stars** |
| Hospital Value-Based Purchasing Participant | 2.317211 | 0.08519 | 27.2005 | < 2.2e-16 | *** |
| Hospital ACO Participant | 0.353294 | 0.100567 | 3.513019 | 1.04E-03 | ** |
| For-profit Hospital | -0.37245 | 0.026412 | -14.1015 | < 2.2e-16 | *** |
| Hospital with Affiliated SNFs | 0.001874 | 0.033235 | 0.057932 | 9.54E-01 |  |
| For-profit SNF | 0.384608 | 0.023354 | 16.46854 | < 2.2e-16 | *** |
| Chain SNF | 0.120943 | 0.01742 | 6.942867 | 1.39E-08 | *** |
| Medicare SNF Bed Count | -0.00481 | 0.00051 | -9.44292 | 3.80E-12 | *** |
| Overall 5-star NH rating | -0.15787 | 0.009468 | -16.6742 | < 2.2e-16 | *** |
| Hospital Rurality: large rural | -0.94133 | 0.10925 | -8.61631 | 5.35E-11 | *** |
| Hospital Rurality: small rural | -0.42881 | 0.099491 | -4.31005 | 9.04E-05 | *** |
| Hospital Rurality: urban | -0.99107 | 0.110605 | -8.96043 | 1.76E-11 | *** |
| SNF Rurality: large rural | -0.00843 | 0.04797 | -0.17582 | 8.61E-01 |  |
| SNF Rurality: small rural | -0.04627 | 0.050932 | -0.9085 | 3.69E-01 |  |
| SNF Rurality: urban | -0.20169 | 0.04977 | -4.05241 | 2.03E-04 | *** |
| Local per capita facility-level COVID-19 | -1.58131 | 1.001476 | -1.57898 | 1.22E-01 |  |
| Treatment*Post PDPM Announcement | 0.876688 | 0.111397 | 7.869956 | 6.21E-10 | *** |
| Significance. codes: 0 ‘***’ 0.001 ‘**’ 0.01 ‘*’ 0.05 ‘.’ 0.1 ‘ ’ 1 | | | | | |

## Table S12. Full Regression Results: Elixhauser, with Profit-Status interaction, among all SNFs

| Full Regression Results: Elixhauser, with Profit-Status interaction, among all SNFs (n= 9,335,052) | | | | | |
| --- | --- | --- | --- | --- | --- |
| **Term** | **Estimate** | **Std. Error** | **t value** | **Pr(>\|t\|)** | **Stars** |
| For-Profit SNF | 0.417004 | 0.035994 | 11.58541 | 0.0000 | *** |
| Hospital Value-Based Purchasing Participant | 2.317042 | 0.085226 | 27.18708 | < 2.2e-16 | *** |
| Hospital ACO Participant | 0.351375 | 0.100816 | 3.485315 | 0.0011 | ** |
| For-profit hospital | -0.37227 | 0.026416 | -14.0927 | < 2.2e-16 | *** |
| Hospital with Affiliated SNFs | 0.001715 | 0.032348 | 0.053004 | 0.9580 |  |
| Chain SNF | 0.119207 | 0.017329 | 6.879174 | 0.0000 | *** |
| Medicare SNF Bed Count | -0.0048 | 0.00051 | -9.40825 | 0.0000 | *** |
| Overall 5-star NH rating | -0.15659 | 0.00946 | -16.5526 | < 2.2e-16 | *** |
| Hospital Rurality: large rural | -0.94064 | 0.10928 | -8.60761 | 0.0000 | *** |
| Hospital Rurality: small rural | -0.42848 | 0.09952 | -4.30545 | 0.0001 | *** |
| Hospital Rurality: urban | -0.9903 | 0.110641 | -8.95057 | 0.0000 | *** |
| SNF Rurality: large rural | -0.00871 | 0.047994 | -0.18148 | 0.8568 |  |
| SNF Rurality: small rural | -0.0472 | 0.050973 | -0.92595 | 0.3595 |  |
| SNF Rurality: urban | -0.20194 | 0.049798 | -4.05528 | 0.0002 | *** |
| Local per capita facility-level COVID-19 | -1.5651 | 1.00221 | -1.56165 | 0.1255 |  |
| Treatment*Post PDPM Announcement | 0.761545 | 0.102373 | 7.438939 | 0.0000 | *** |
| Treatment* SNF Profit Status | -0.34862 | 0.082209 | -4.24069 | 0.0001 | *** |
| Post PDPM Announcement* SNF Profit Status | -0.01737 | 0.033703 | -0.51533 | 0.6089 |  |
| Treatment*Post PDPM Announcement*SNF Profit Status | 0.155672 | 0.047731 | 3.26146 | 0.0021 | ** |
| Significance. codes: 0 ‘***’ 0.001 ‘**’ 0.01 ‘*’ 0.05 ‘.’ 0.1 ‘ ’ 1 | | | | | |

## Table S13. Full Regression Results: Elixhauser, among all For-Profit SNFs

| Full Regression Results: Elixhauser, among all SNFs (n= 6,801,105) | | | | | |
| --- | --- | --- | --- | --- | --- |
| **Term** | **Estimate** | **Std. Error** | **t value** | **Pr(>\|t\|)** | **Stars** |
| Hospital Value-Based Purchasing Participant | 2.427344 | 0.092241 | 26.3153 | < 2.2e-16 | *** |
| Hospital ACO Participant | 0.365175 | 0.11299 | 3.231917 | 2.33E-03 | ** |
| For-profit Hospital | -0.33498 | 0.02858 | -11.7207 | 4.00E-15 | *** |
| Hospital with Affiliated SNFs | 0.021645 | 0.037703 | 0.574084 | 5.69E-01 |  |
| Chain SNF | 0.106032 | 0.020665 | 5.131048 | 6.25E-06 | *** |
| Medicare SNF Bed Count | -0.00566 | 0.000582 | -9.71879 | 1.60E-12 | *** |
| Overall 5-star NH rating | -0.12619 | 0.010082 | -12.5162 | 4.27E-16 | *** |
| Hospital Rurality: large rural | -0.86188 | 0.124805 | -6.90577 | 1.57E-08 | *** |
| Hospital Rurality: small rural | -0.4267 | 0.117727 | -3.62446 | 7.47E-04 | *** |
| Hospital Rurality: urban | -0.89246 | 0.126302 | -7.06605 | 9.15E-09 | *** |
| SNF Rurality: large rural | 0.038392 | 0.059239 | 0.648081 | 5.20E-01 |  |
| SNF Rurality: small rural | 0.00514 | 0.061996 | 0.082915 | 9.34E-01 |  |
| SNF Rurality: urban | -0.10906 | 0.058136 | -1.876 | 6.73E-02 | . |
| Local per capita facility-level COVID-19 | -1.81542 | 1.083331 | -1.67577 | 1.01E-01 |  |
| Treatment*Post PDPM Announcement | 0.920511 | 0.118196 | 7.788024 | 8.15E-10 | *** |
| Significance. codes: 0 ‘***’ 0.001 ‘**’ 0.01 ‘*’ 0.05 ‘.’ 0.1 ‘ ’ 1 | | | | | |

## Table S14. Full Regression Results: Elixhauser, among all Not-for-Profit SNFs

| Full Regression Results: Elixhauser, among all SNFs (n= 2,533,947) | | | | | |
| --- | --- | --- | --- | --- | --- |
| **Term** | **Estimate** | **Std. Error** | **t value** | **Pr(>\|t\|)** | **Stars** |
| Hospital Value-Based Purchasing Participant | 1.872891 | 0.092533 | 20.24019 | < 2.2e-16 | *** |
| Hospital ACO Participant | 0.338701 | 0.128896 | 2.62772 | 1.18E-02 | * |
| For-profit Hospital | -0.50415 | 0.049695 | -10.1449 | 4.28E-13 | *** |
| Hospital with Affiliated SNFs | -0.06412 | 0.055783 | -1.14952 | 2.57E-01 |  |
| Chain SNF | 0.129975 | 0.035103 | 3.702698 | 5.91E-04 | *** |
| Medicare SNF Bed Count | -0.00338 | 0.000836 | -4.04106 | 2.10E-04 | *** |
| Overall 5-star NH rating | -0.23354 | 0.015161 | -15.4041 | < 2.2e-16 | *** |
| Hospital Rurality: large rural | -0.97979 | 0.179022 | -5.47301 | 1.99E-06 | *** |
| Hospital Rurality: small rural | -0.37046 | 0.168248 | -2.20187 | 3.30E-02 | * |
| Hospital Rurality: urban | -1.06481 | 0.174139 | -6.11469 | 2.29E-07 | *** |
| SNF Rurality: large rural | -0.07147 | 0.108153 | -0.66085 | 5.12E-01 |  |
| SNF Rurality: small rural | -0.1275 | 0.100558 | -1.26789 | 2.12E-01 |  |
| SNF Rurality: urban | -0.3217 | 0.108305 | -2.97034 | 4.80E-03 | ** |
| Local per capita facility-level COVID-19 | -1.16455 | 1.249059 | -0.93234 | 3.56E-01 |  |
| Treatment*Post PDPM Announcement | 0.752081 | 0.101518 | 7.408351 | 2.89E-09 | *** |
| Significance. codes: 0 ‘***’ 0.001 ‘**’ 0.01 ‘*’ 0.05 ‘.’ 0.1 ‘ ’ 1 | | | | | |

## Table S15. Full Regression Results: Probability of Documenting Chronic Pulmonary Disease

| Full Regression Results: Chronic Pulmonary Disease (n=9,330,179) | | | | | |
| --- | --- | --- | --- | --- | --- |
| **Term** | **Estimate** | **Std. Error** | **t value** | **Pr(>\|t\|)** | **Stars** |
| Hospital Value-Based Purchasing Participant | 0.160821 | 0.010748 | 14.96264 | < 2.2e-16 | *** |
| Hospital ACO Participant | -0.0103 | 0.028856 | -0.35689 | 0.72 |  |
| For-profit Hospital | 0.018756 | 0.005607 | 3.345298 | 0.00 | *** |
| Hospital with Affiliated SNFs | 0.002018 | 0.007744 | 0.260589 | 0.79 |  |
| For-profit SNF | 0.101172 | 0.004434 | 22.81502 | < 2.2e-16 | *** |
| Chain SNF | 0.017707 | 0.003715 | 4.766224 | 0.00 | *** |
| Medicare SNF Bed Count | -0.0009 | 0.000093 | -9.5804 | < 2.2e-16 | *** |
| Overall 5-star NH rating | -0.03905 | 0.001581 | -24.703 | < 2.2e-16 | *** |
| Hospital Rurality: large rural | -0.05483 | 0.02626 | -2.08794 | 0.04 | * |
| Hospital Rurality: small rural | 0.001434 | 0.026872 | 0.053356 | 0.96 |  |
| Hospital Rurality: urban | -0.21382 | 0.027285 | -7.83622 | 0.00 | *** |
| SNF Rurality: large rural | -0.02785 | 0.011985 | -2.32335 | 0.02 | * |
| SNF Rurality: small rural | -0.03116 | 0.013335 | -2.3366 | 0.02 | * |
| SNF Rurality: urban | -0.06837 | 0.012032 | -5.68214 | 0.00 | *** |
| Local per capita facility-level COVID-19 | 0.008533 | 0.178214 | 0.04788 | 0.96 |  |
| Treatment×Post PDPM Announcement | 0.203818 | 0.025777 | 7.907047 | 0.00 | *** |
| Significance. codes: 0 ‘***’ 0.001 ‘**’ 0.01 ‘*’ 0.05 ‘.’ 0.1 ‘ ’ 1  Note: reported coefficients reflect log-odds from logistic regression models; marginal effects for Treatment×Post PDPM Announcement are presented in Main Table 2 for interpretability. | | | | | |

## Table S16. Full Regression Results: Probability of Documenting Complicated Diabetes

| Full Regression Results: Complicated Diabetes (n=9,309,328) | | | | | |
| --- | --- | --- | --- | --- | --- |
| **Term** | **Estimate** | **Std. Error** | **t value** | **Pr(>\|t\|)** | **Stars** |
| Hospital Value-Based Purchasing Participant | 0.272195 | 0.015033 | 18.10672 | < 2.2e-16 | *** |
| Hospital ACO Participant | 0.116881 | 0.028424 | 4.112038 | 3.92E-05 | *** |
| For-profit Hospital | -0.06786 | 0.008467 | -8.01454 | 1.11E-15 | *** |
| Hospital with Affiliated SNFs | 0.004935 | 0.010518 | 0.469188 | 6.39E-01 |  |
| For-profit SNF | 0.176684 | 0.00637 | 27.73734 | < 2.2e-16 | *** |
| Chain SNF | 0.041612 | 0.004558 | 9.128627 | < 2.2e-16 | *** |
| Medicare SNF Bed Count | -0.00188 | 0.00013 | -14.4645 | < 2.2e-16 | *** |
| Overall 5-star NH rating | -0.0678 | 0.002379 | -28.5019 | < 2.2e-16 | *** |
| Hospital Rurality: large rural | -0.09756 | 0.02539 | -3.84237 | 1.22E-04 | *** |
| Hospital Rurality: small rural | -0.07135 | 0.027422 | -2.60177 | 9.27E-03 | ** |
| Hospital Rurality: urban | 0.001999 | 0.026971 | 0.074129 | 9.41E-01 |  |
| SNF Rurality: large rural | 0.026969 | 0.013136 | 2.053006 | 4.01E-02 | * |
| SNF Rurality: small rural | 0.012855 | 0.013749 | 0.93502 | 3.50E-01 |  |
| SNF Rurality: urban | -0.04545 | 0.013317 | -3.41254 | 6.44E-04 | *** |
| Local per capita facility-level COVID-19 | 0.637323 | 0.214865 | 2.966157 | 3.02E-03 | ** |
| Treatment×Post PDPM Announcement | 0.316904 | 0.032919 | 9.62673 | < 2.2e-16 | *** |
| Significance. codes: 0 ‘***’ 0.001 ‘**’ 0.01 ‘*’ 0.05 ‘.’ 0.1 ‘ ’ 1  Note: reported coefficients reflect log-odds from logistic regression models; marginal effects for Treatment×Post PDPM Announcement are presented in Main Table 2 for interpretability. | | | | | |

## Table S17. Full Regression Results: Probability of Documenting Heart Failure

| Full Regression Results: Heart Failure (n=9,329,980) | | | | | |
| --- | --- | --- | --- | --- | --- |
| **Term** | **Estimate** | **Std. Error** | **t value** | **Pr(>\|t\|)** | **Stars** |
| Hospital Value-Based Purchasing Participant | 0.429516 | 0.018322 | 23.44249 | < 2.2e-16 | *** |
| Hospital ACO Participant | 0.023467 | 0.031571 | 0.74332 | 4.57E-01 |  |
| For-profit Hospital | -0.03547 | 0.006925 | -5.12255 | 3.01E-07 | *** |
| Hospital with Affiliated SNFs | 0.01094 | 0.009722 | 1.12527 | 2.60E-01 |  |
| For-profit SNF | 0.011733 | 0.004386 | 2.675277 | 7.47E-03 | ** |
| Chain SNF | 0.006906 | 0.002997 | 2.304071 | 2.12E-02 | * |
| Medicare SNF Bed Count | -0.00044 | 0.000089 | -4.87887 | 1.07E-06 | *** |
| Overall 5-star NH rating | -0.00249 | 0.001702 | -1.46025 | 1.44E-01 |  |
| Hospital Rurality: large rural | -0.21631 | 0.027643 | -7.82511 | 5.07E-15 | *** |
| Hospital Rurality: small rural | -0.0759 | 0.028189 | -2.69242 | 7.09E-03 | ** |
| Hospital Rurality: urban | -0.31515 | 0.028331 | -11.1236 | < 2.2e-16 | *** |
| SNF Rurality: large rural | 0.00579 | 0.011085 | 0.522313 | 6.01E-01 |  |
| SNF Rurality: small rural | 0.002496 | 0.011663 | 0.214055 | 8.31E-01 |  |
| SNF Rurality: urban | -0.03732 | 0.011472 | -3.25347 | 1.14E-03 | ** |
| Local per capita facility-level COVID-19 | -0.55599 | 0.195797 | -2.85961 | 4.24E-03 | ** |
| Treatment×Post PDPM Announcement | 0.135873 | 0.015709 | 8.649135 | < 2.2e-16 | *** |
| Significance. codes: 0 ‘***’ 0.001 ‘**’ 0.01 ‘*’ 0.05 ‘.’ 0.1 ‘ ’ 1  Note: reported coefficients reflect log-odds from logistic regression models; marginal effects for Treatment×Post PDPM Announcement are presented in Main Table 2 for interpretability. | | | | | |

## Table S18. Full Regression Results: Probability of Documenting Obesity

| Full Regression Results: Obesity (n=9,219,075) | | | | | |
| --- | --- | --- | --- | --- | --- |
| **Term** | **Estimate** | **Std. Error** | **t value** | **Pr(>\|t\|)** | **Stars** |
| Hospital Value-Based Purchasing Participant | 0.184725 | 0.017709 | 10.43129 | < 2.2e-16 | *** |
| Hospital ACO Participant | 0.060253 | 0.029023 | 2.076047 | 3.79E-02 | * |
| For-profit Hospital | -0.07892 | 0.009571 | -8.24603 | < 2.2e-16 | *** |
| Hospital with Affiliated SNFs | -0.02281 | 0.013718 | -1.66277 | 9.64E-02 | . |
| For-profit SNF | 0.13855 | 0.005988 | 23.13753 | < 2.2e-16 | *** |
| Chain SNF | 0.043295 | 0.004501 | 9.619862 | < 2.2e-16 | *** |
| Medicare SNF Bed Count | -0.0007 | 0.000121 | -5.77874 | 7.53E-09 | *** |
| Overall 5-star NH rating | -0.04051 | 0.002163 | -18.7273 | < 2.2e-16 | *** |
| Hospital Rurality: large rural | 0.143236 | 0.038665 | 3.704555 | 2.12E-04 | *** |
| Hospital Rurality: small rural | 0.111194 | 0.041116 | 2.704414 | 6.84E-03 | ** |
| Hospital Rurality: urban | 0.20411 | 0.039041 | 5.228148 | 1.71E-07 | *** |
| SNF Rurality: large rural | 0.014899 | 0.015046 | 0.990263 | 3.22E-01 |  |
| SNF Rurality: small rural | 0.005304 | 0.016108 | 0.329294 | 7.42E-01 |  |
| SNF Rurality: urban | -0.06704 | 0.01485 | -4.51445 | 6.35E-06 | *** |
| Local per capita facility-level COVID-19 | 1.044523 | 0.294679 | 3.544616 | 3.93E-04 | *** |
| Treatment×Post PDPM Announcement | 0.645924 | 0.059518 | 10.85259 | < 2.2e-16 | *** |
| Significance. codes: 0 ‘***’ 0.001 ‘**’ 0.01 ‘*’ 0.05 ‘.’ 0.1 ‘ ’ 1  Note: reported coefficients reflect log-odds from logistic regression models; marginal effects for Treatment×Post PDPM Announcement are presented in Main Table 2 for interpretability. | | | | | |

## Table S19. Full Regression Results: Probability of Documenting Weight Loss

| Full Regression Results: Weight Loss (n=9,230,160) | | | | | |
| --- | --- | --- | --- | --- | --- |
| **Term** | **Estimate** | **Std. Error** | **t value** | **Pr(>\|t\|)** | **Stars** |
| Hospital Value-Based Purchasing Participant | 0.305719 | 0.024065 | 12.70402 | < 2.2e-16 | *** |
| Hospital ACO Participant | 0.034938 | 0.023569 | 1.482392 | 1.38E-01 |  |
| For-profit Hospital | -0.02982 | 0.014513 | -2.0547 | 3.99E-02 | * |
| Hospital with Affiliated SNFs | -0.00966 | 0.019473 | -0.49594 | 6.20E-01 |  |
| For-profit SNF | 0.125842 | 0.007088 | 17.75381 | < 2.2e-16 | *** |
| Chain SNF | 0.042987 | 0.005297 | 8.114551 | 4.88E-16 | *** |
| Medicare SNF Bed Count | -0.00111 | 0.000121 | -9.12964 | < 2.2e-16 | *** |
| Overall 5-star NH rating | -0.05989 | 0.002758 | -21.7134 | < 2.2e-16 | *** |
| Hospital Rurality: large rural | -0.01429 | 0.048101 | -0.29713 | 7.66E-01 |  |
| Hospital Rurality: small rural | -0.02605 | 0.050365 | -0.51719 | 6.05E-01 |  |
| Hospital Rurality: urban | -0.07517 | 0.049421 | -1.52097 | 1.28E-01 |  |
| SNF Rurality: large rural | -0.01972 | 0.01512 | -1.30419 | 1.92E-01 |  |
| SNF Rurality: small rural | -0.02114 | 0.016387 | -1.28995 | 1.97E-01 |  |
| SNF Rurality: urban | 0.034056 | 0.014605 | 2.331768 | 1.97E-02 | * |
| Local per capita facility-level COVID-19 | 1.886083 | 0.531177 | 3.550765 | 3.84E-04 | *** |
| Treatment×Post PDPM Announcement | 0.933758 | 0.093949 | 9.938991 | < 2.2e-16 | *** |
| Significance. codes: 0 ‘***’ 0.001 ‘**’ 0.01 ‘*’ 0.05 ‘.’ 0.1 ‘ ’ 1  Note: reported coefficients reflect log-odds from logistic regression models; marginal effects for Treatment×Post PDPM Announcement are presented in Main Table 2 for interpretability. | | | | | |

## Figure S1. Event Study: Diagnosis Count


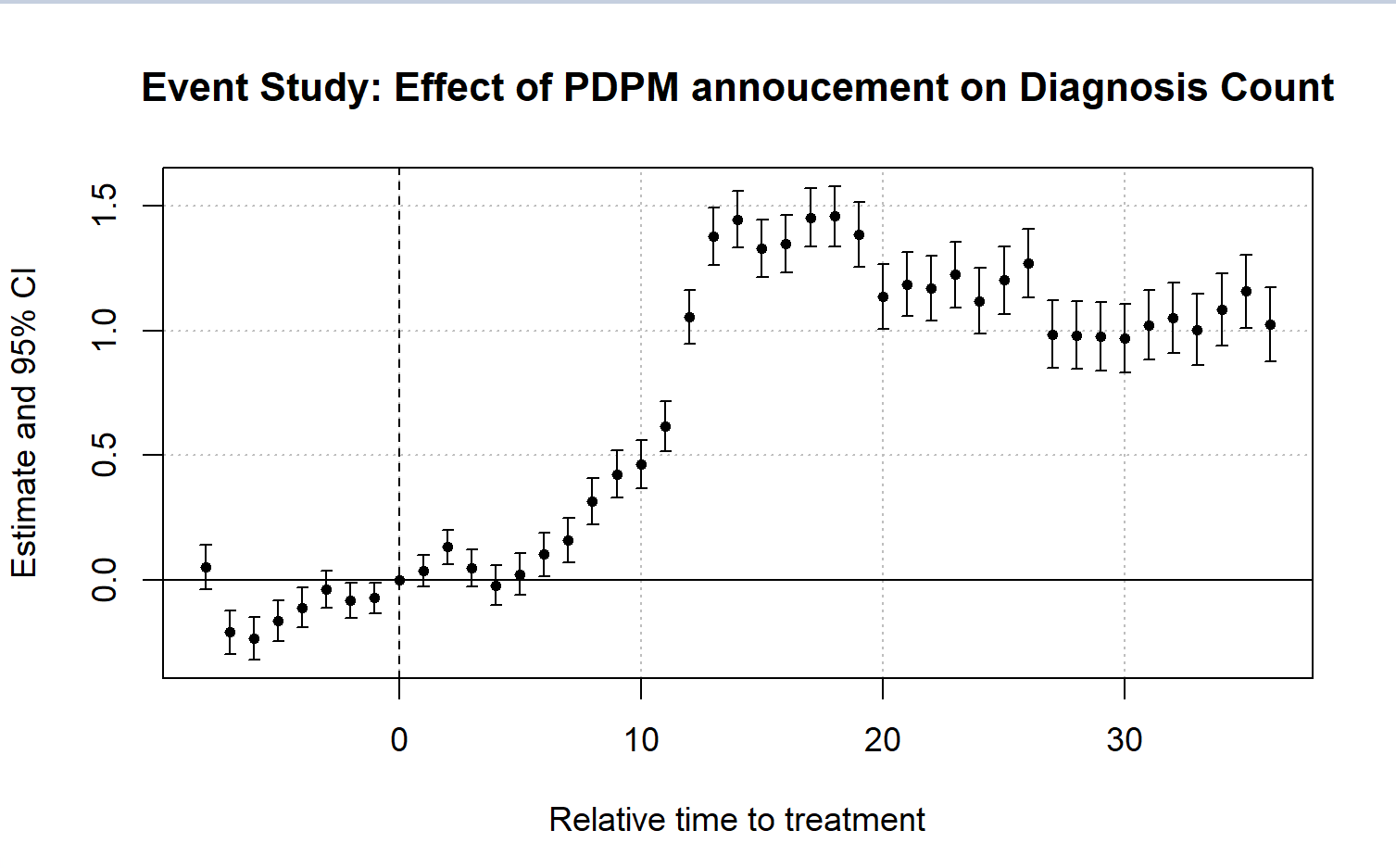


Event-study estimates of changes in the monthly number of diagnoses recorded on SNF (treatment) and Hospital (control) claims, with event time defined relative to the Patient-Driven Payment Model (PDPM) announcement, defined as the Final Rule effective date (October 1, 2018). Time is measured by the year–month of SNF admission, and all models adjust for facility fixed effects.

## Figure S2. Event Study: Elixhauser


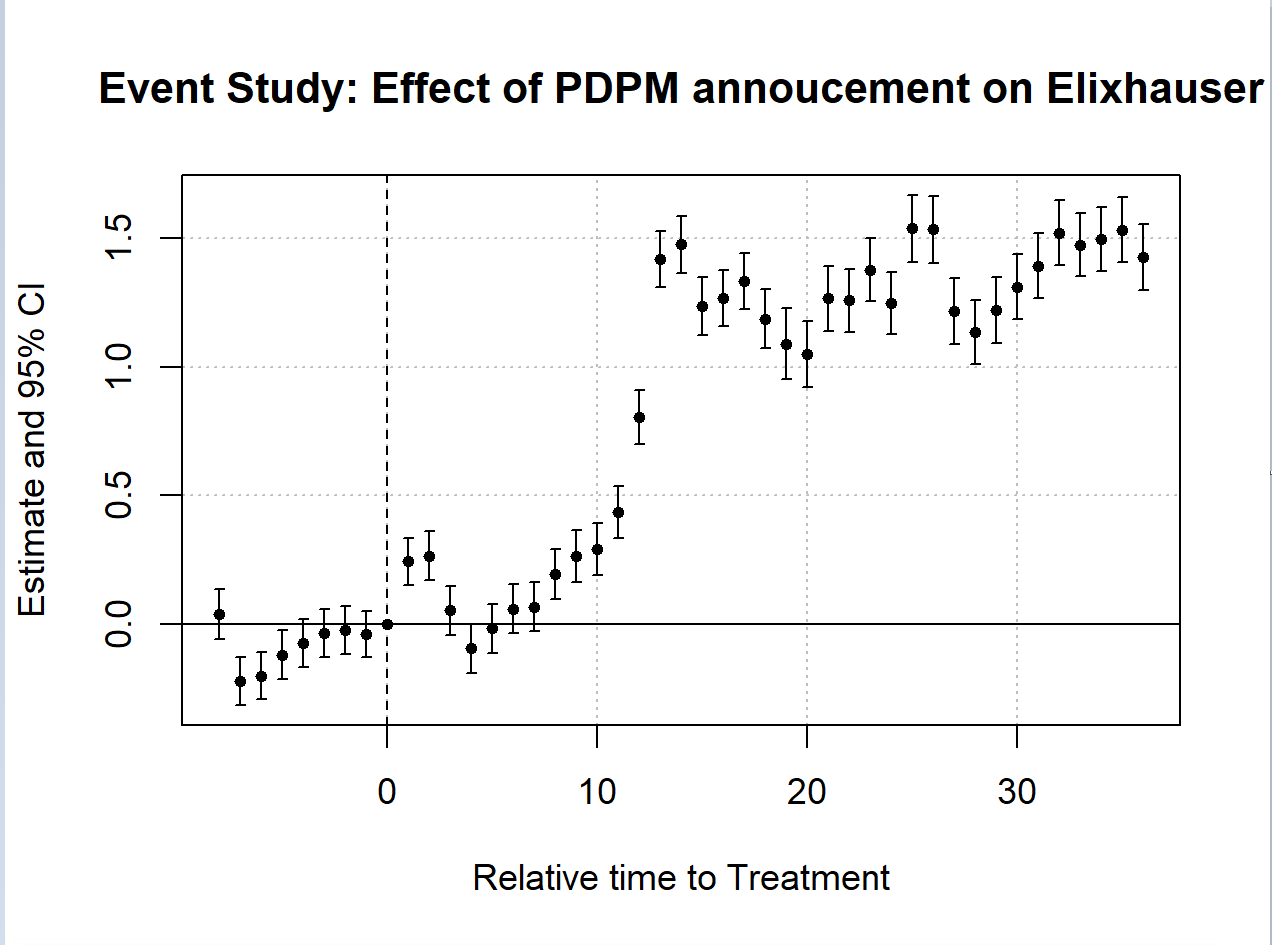


Event-study estimates of changes in the monthly Elixhauser scores derived from SNF (treatment) and Hospital (control) claims, with event time defined relative to the Patient-Driven Payment Model (PDPM) announcement, defined as the Final Rule effective date (October 1, 2018). Time is measured by the year–month of SNF admission, and all models adjust for facility fixed effects.

## Figure S3.a Unadjusted Chronic Pulmonary Disease


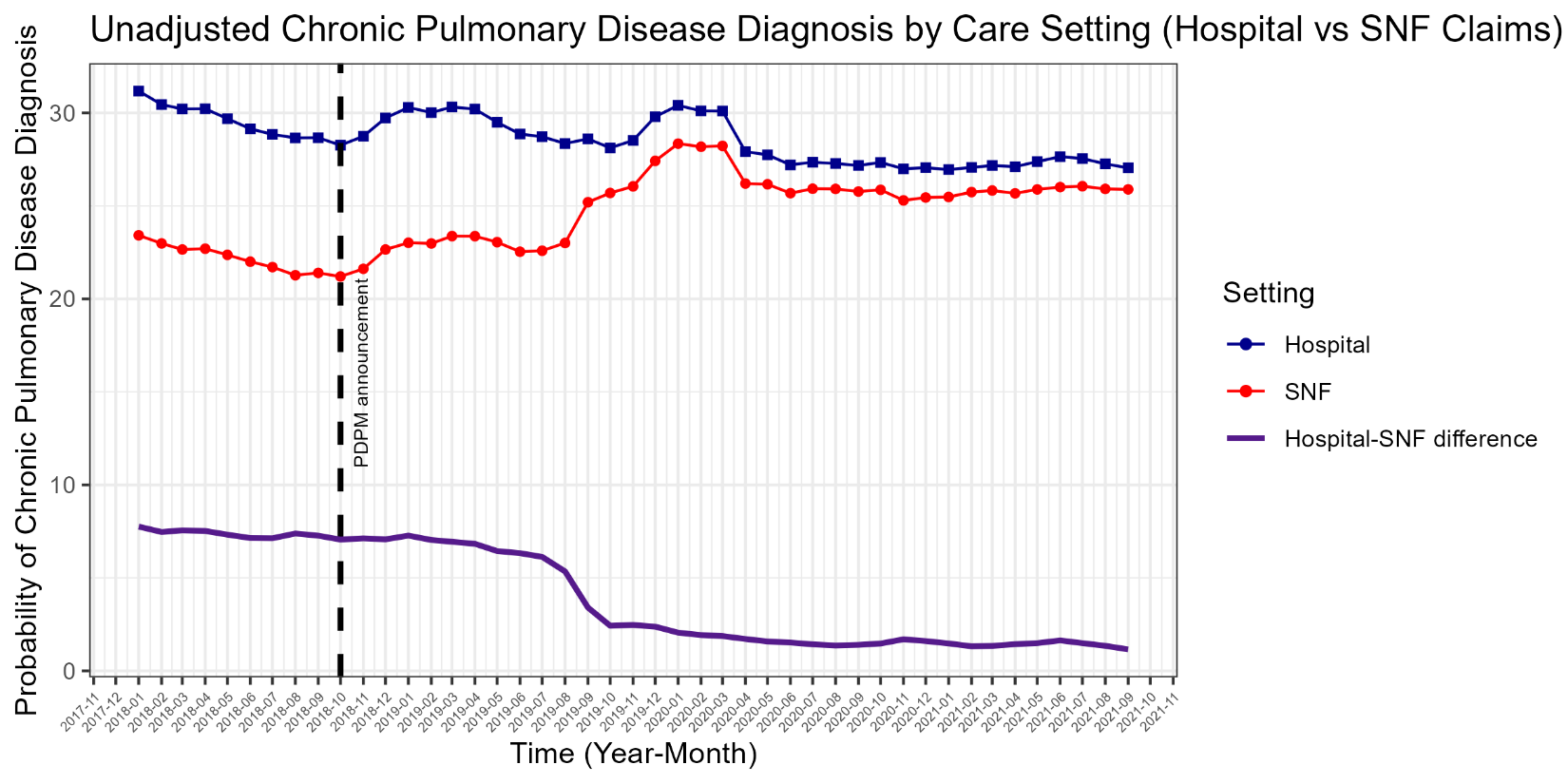


Unadjusted monthly mean probability of chronic pulmonary disease diagnosis among all hospitalized patients (blue line) and those discharged to a SNF (red line), along with the difference in probability between settings (purple line), before and after the Patient-Driven Payment Model (PDPM) announcement (vertical dashed line), defined as the Final Rule effective date (October 1, 2018). Time is indexed by the month of hospital admission.

## Figure S3.b. Event Study: Chronic Pulmonary Disease


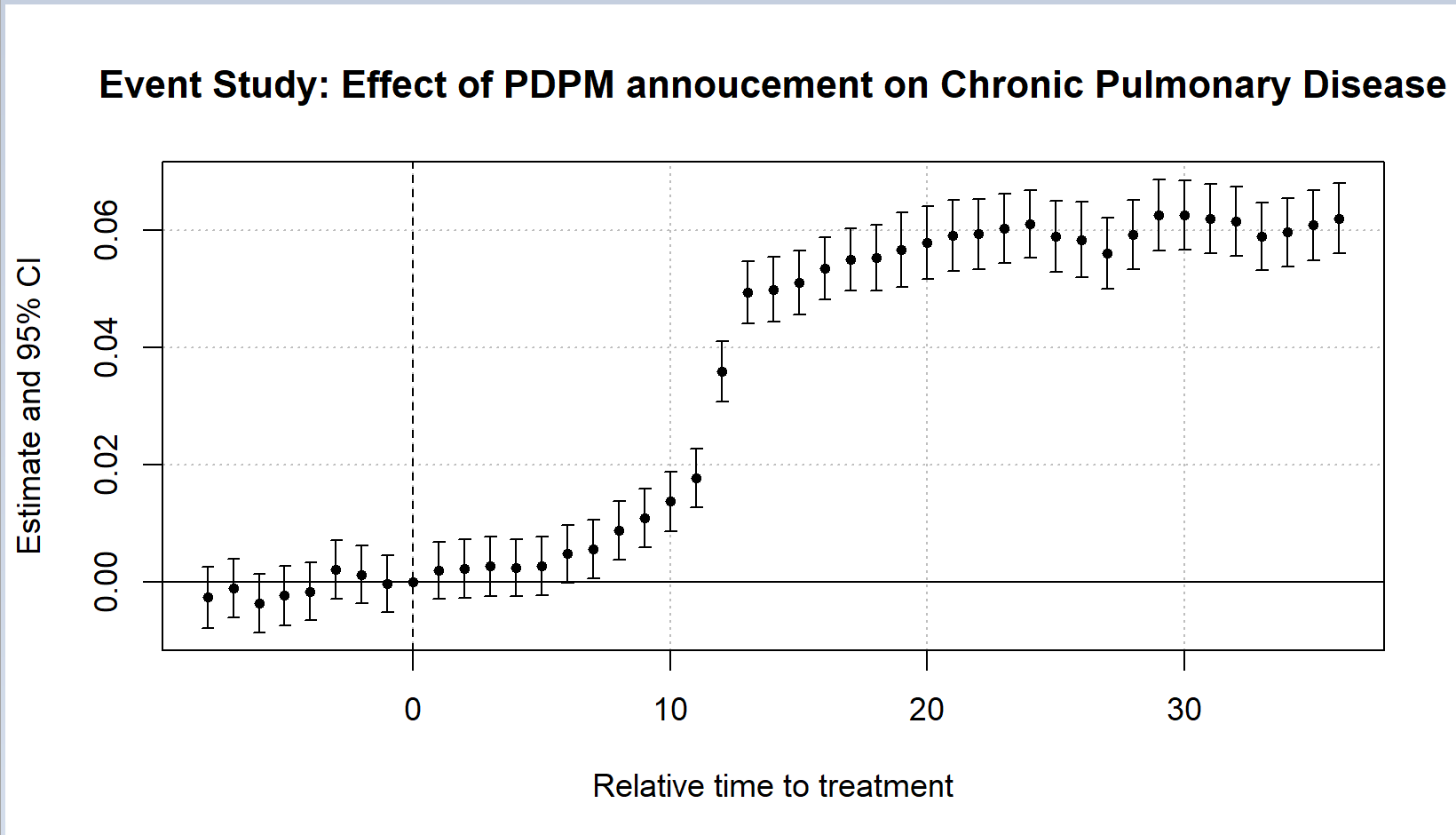


Event-study estimates of changes in the monthly prevalence of chronic pulmonary disease diagnosis derived from SNF (treatment) and Hospital (control) claims, with event time defined relative to the Patient-Driven Payment Model (PDPM) announcement, defined as the Final Rule effective date (October 1, 2018). Time is measured by the year–month of SNF admission, and all models adjust for facility fixed effects.

## Figure S4.a. Unadjusted Complicated Diabetes


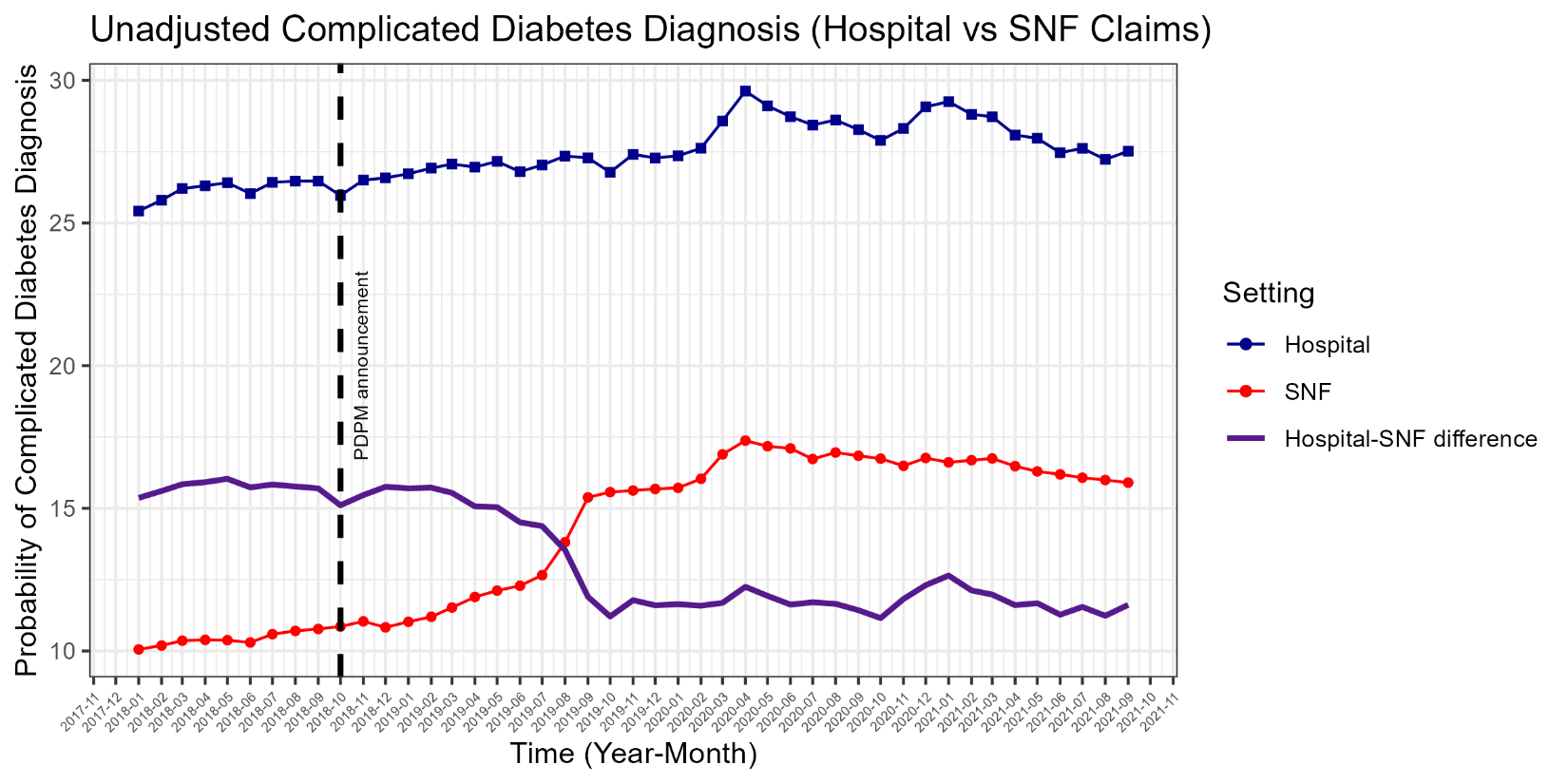


Unadjusted monthly mean probability of complicated diabetes diagnosis among all hospitalized patients (blue line) and those discharged to a SNF (red line), along with the difference in probability between settings (purple line), before and after the Patient-Driven Payment Model (PDPM) announcement (vertical dashed line), defined as the Final Rule effective date (October 1, 2018). Time is indexed by the month of hospital admission.

## Figure S4.b. Event Study: Complicated Diabetes


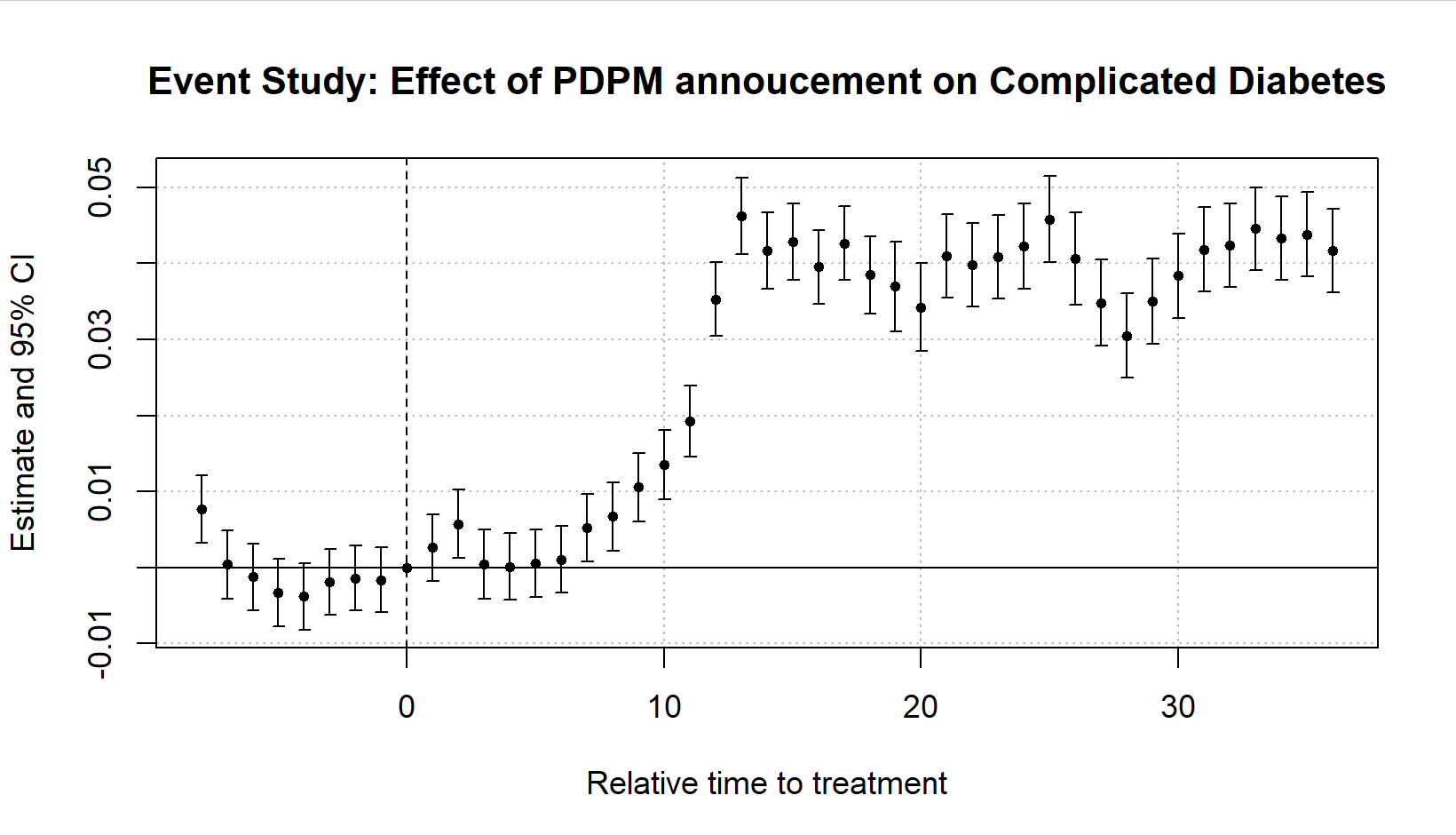


Event-study estimates of changes in the monthly prevalence of complicated diabetes diagnosis derived from SNF (treatment) and Hospital (control) claims, with event time defined relative to the Patient-Driven Payment Model (PDPM) announcement, defined as the Final Rule effective date (October 1, 2018). Time is measured by the year–month of SNF admission, and all models adjust for facility fixed effects.

## Figure S5.a. Unadjusted Heart Failure


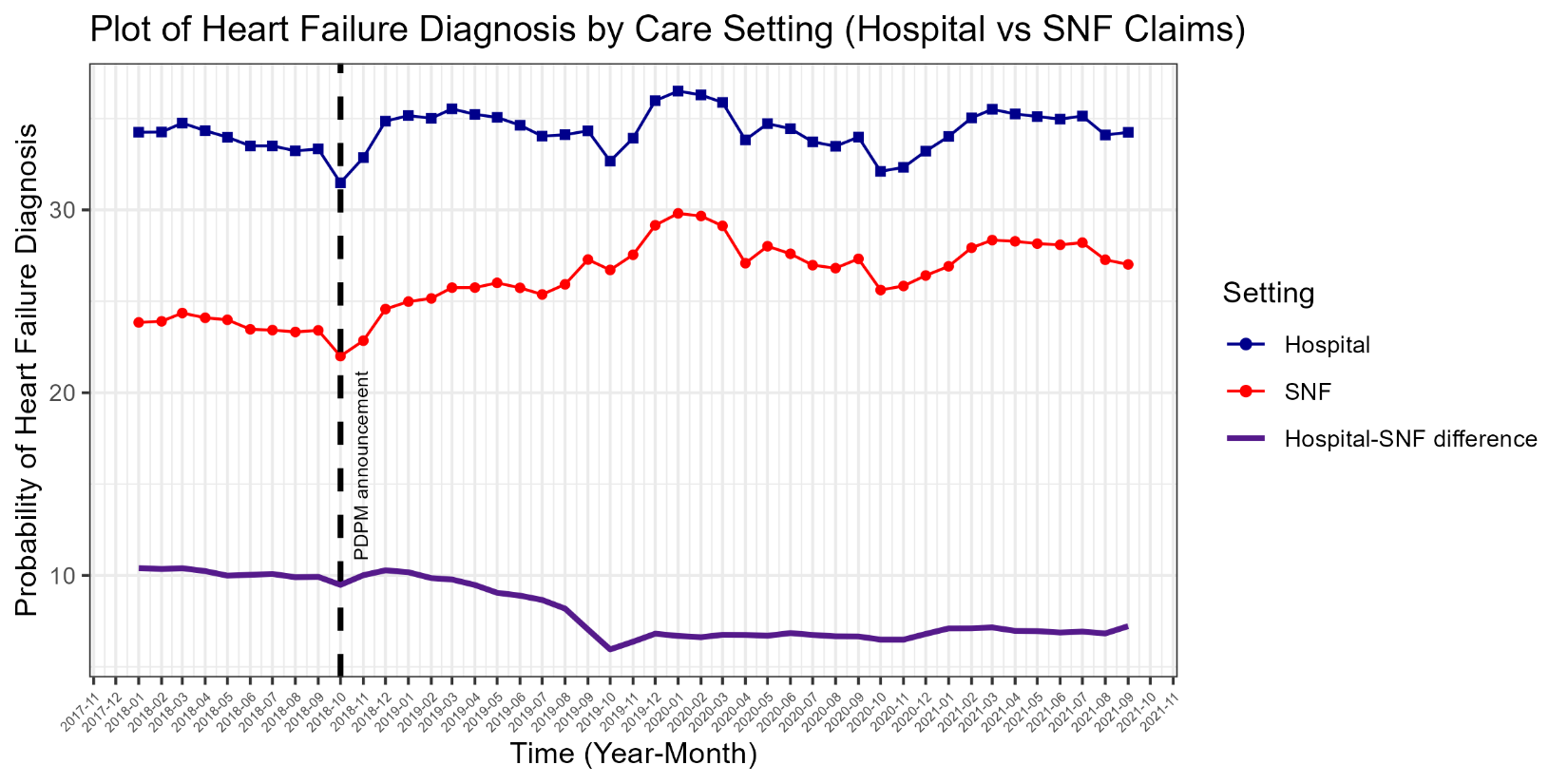


Unadjusted monthly mean probability of heart failure diagnosis among all hospitalized patients (blue line) and those discharged to a SNF (red line), along with the difference in probability between settings (purple line), before and after the Patient-Driven Payment Model (PDPM) announcement (vertical dashed line), defined as the Final Rule effective date (October 1, 2018). Time is indexed by the month of hospital admission.

## Figure S5.b. Event Study: Heart Failure


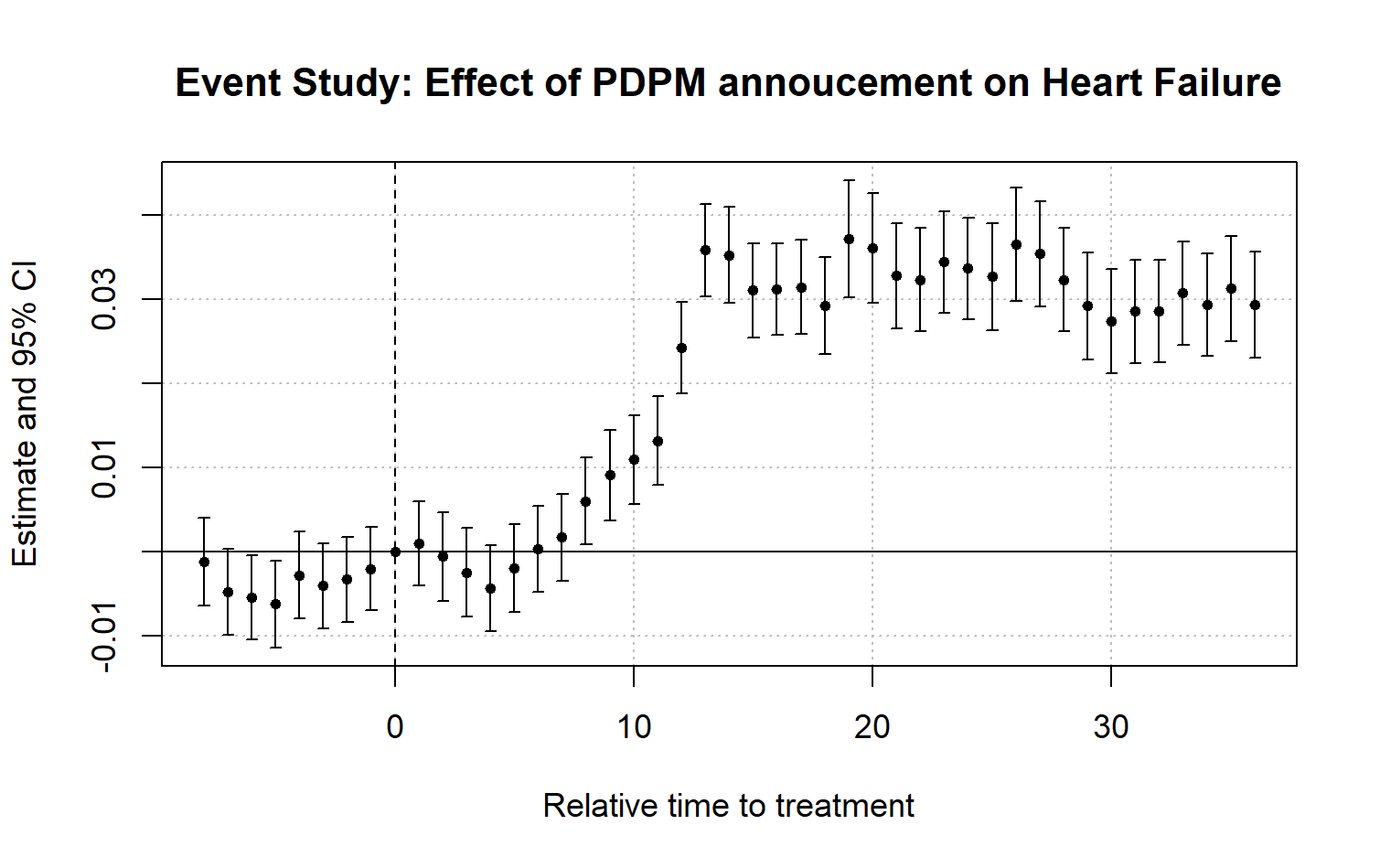


Event-study estimates of changes in the monthly prevalence of heart failure diagnosis derived from SNF (treatment) and Hospital (control) claims, with event time defined relative to the Patient-Driven Payment Model (PDPM) announcement, defined as the Final Rule effective date (October 1, 2018). Time is measured by the year–month of SNF admission, and all models adjust for facility fixed effects.

## Figure S6.a. Unadjusted Obesity


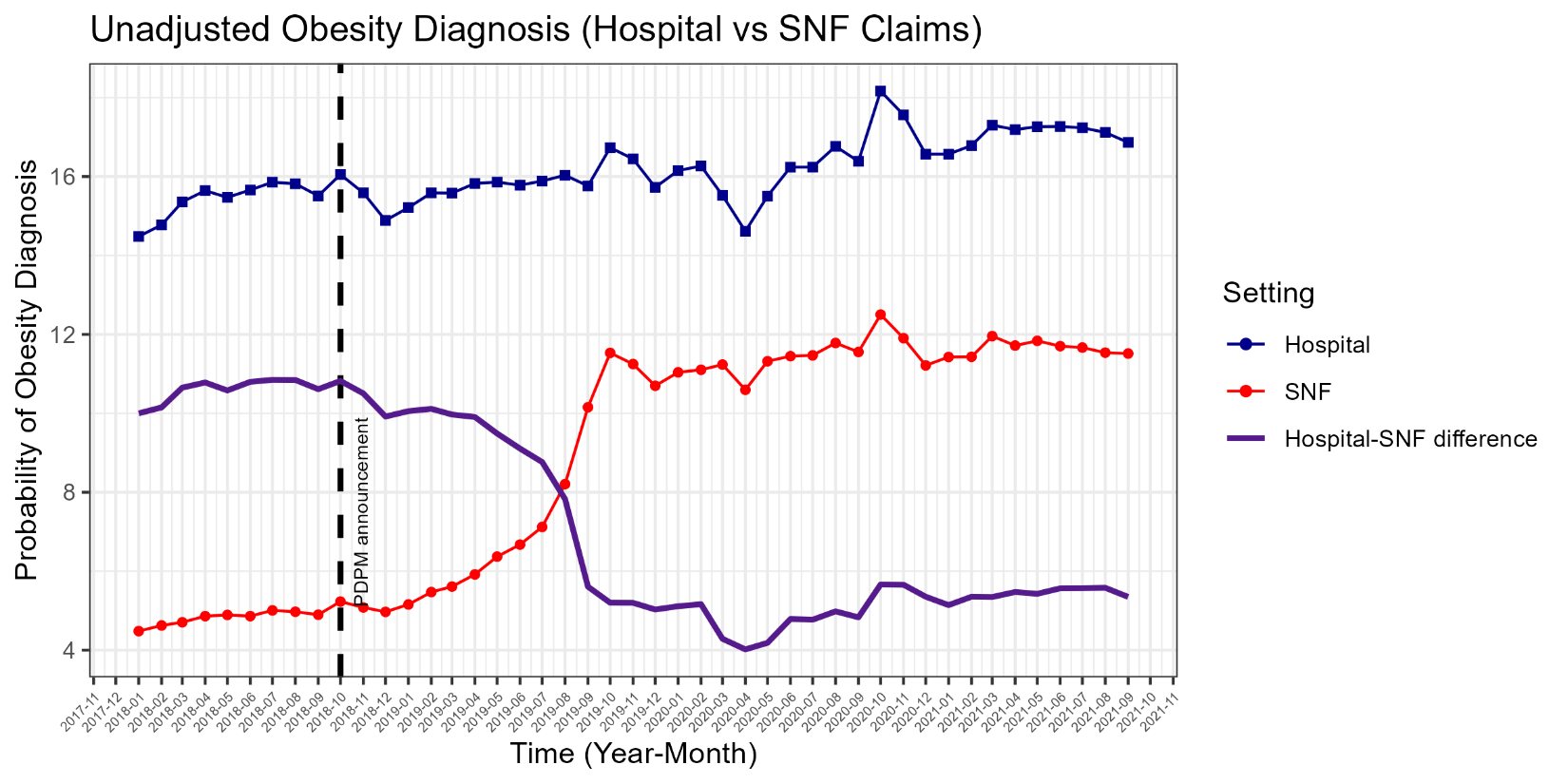


Unadjusted monthly mean probability of obesity diagnosis among all hospitalized patients (blue line) and those discharged to a SNF (red line), along with the difference in probability between settings (purple line), before and after the Patient-Driven Payment Model (PDPM) announcement (vertical dashed line), defined as the Final Rule effective date (October 1, 2018). Time is indexed by the month of hospital admission.

## Figure S6.b Event Study: Obesity


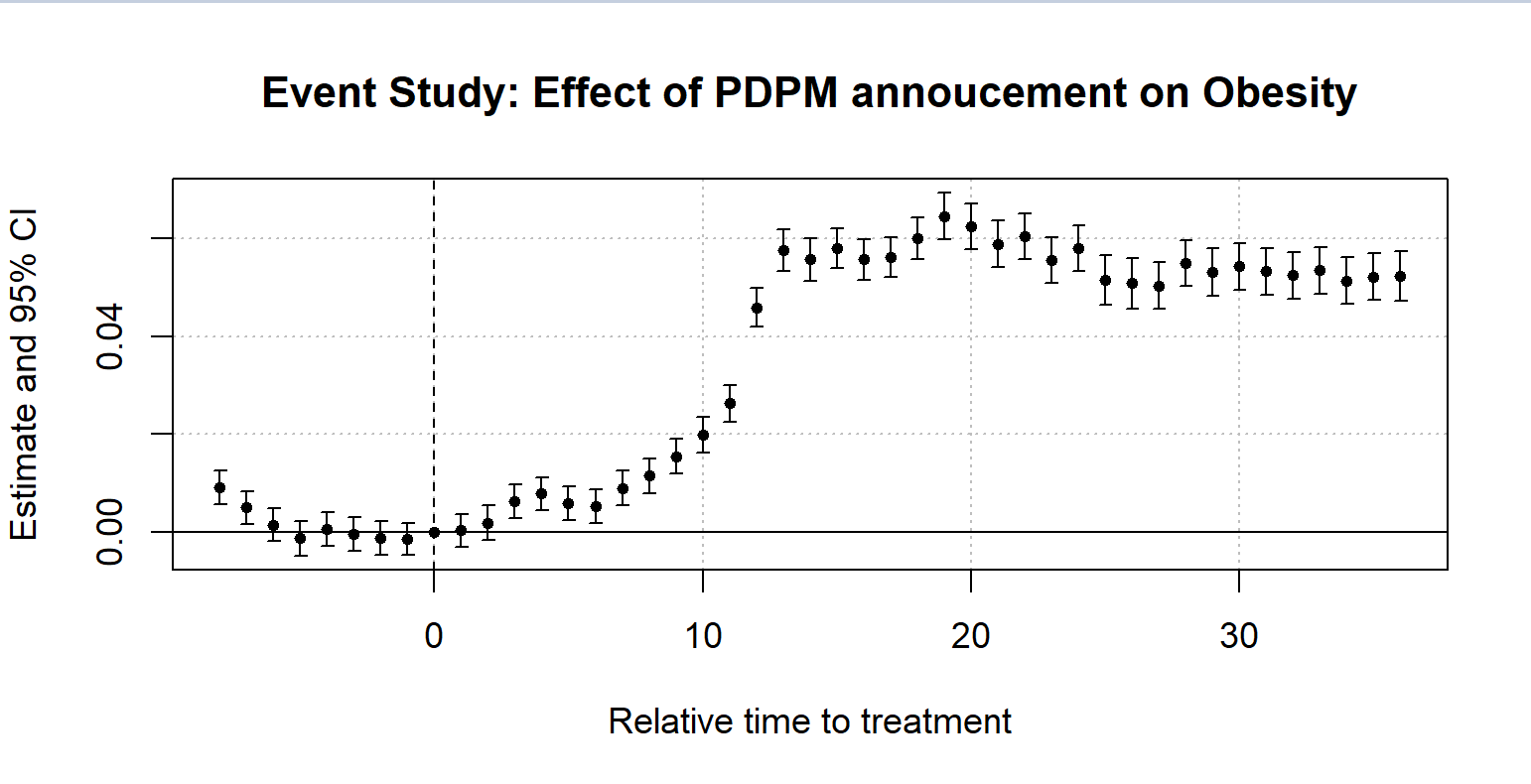


Event-study estimates of changes in the monthly prevalence of obesity diagnosis derived from SNF (treatment) and Hospital (control) claims, with event time defined relative to the Patient-Driven Payment Model (PDPM) announcement, defined as the Final Rule effective date (October 1, 2018). Time is measured by the year–month of SNF admission, and all models adjust for facility fixed effects.

## Figure S7.a Unadjusted Weight loss


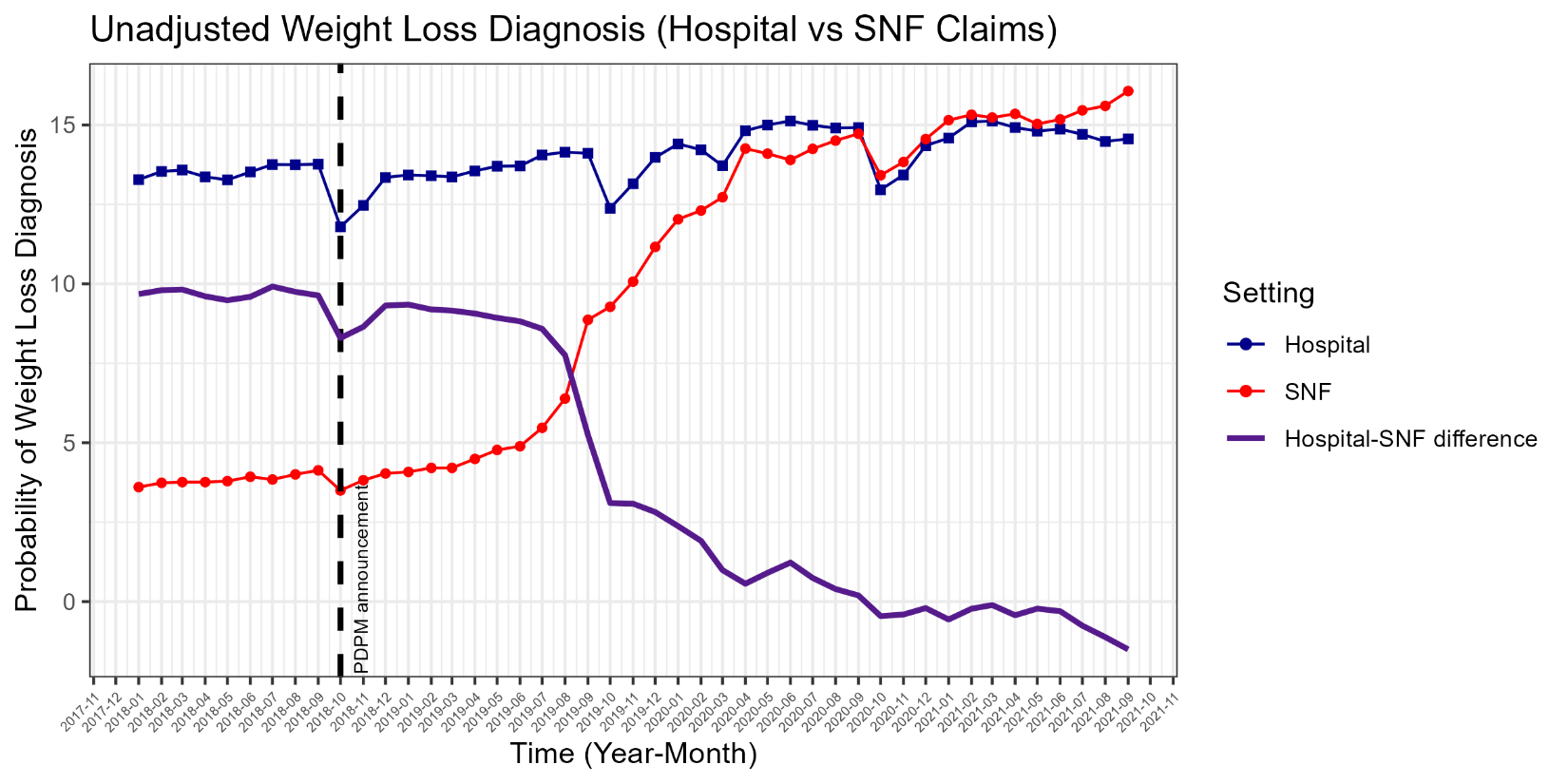


Unadjusted monthly mean probability of weight loss diagnosis among all hospitalized patients (blue line) and those discharged to a SNF (red line), along with the difference in probability between settings (purple line), before and after the Patient-Driven Payment Model (PDPM) announcement (vertical dashed line), defined as the Final Rule effective date (October 1, 2018). Time is indexed by the month of hospital admission.

## Figure S7.b Event Study: Weight loss


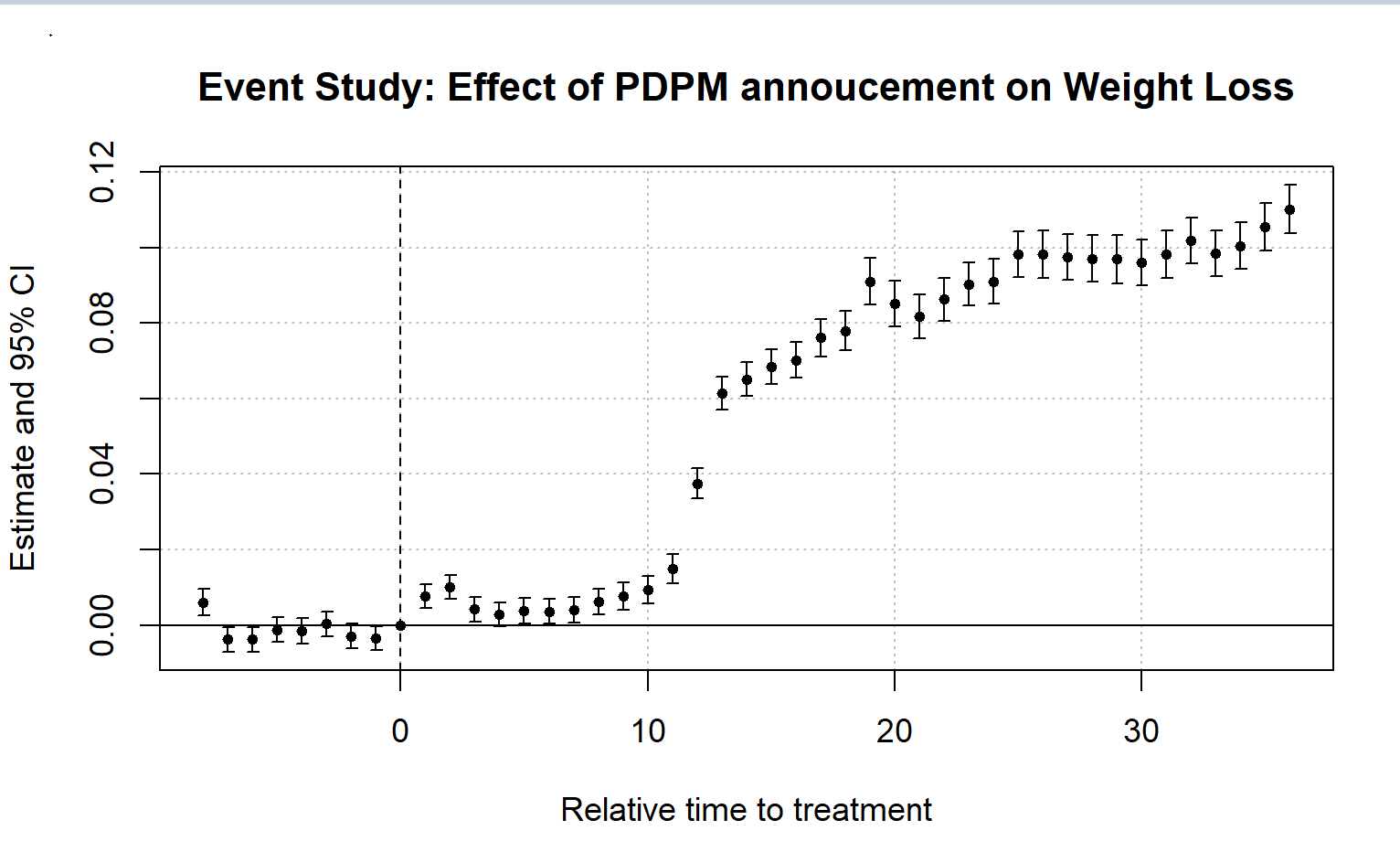


Event-study estimates of changes in the monthly prevalence of weight loss diagnosis derived from SNF (treatment) and Hospital (control) claims, with event time defined relative to the Patient-Driven Payment Model (PDPM) announcement, defined as the Final Rule effective date (October 1, 2018). Time is measured by the year–month of SNF admission, and all models adjust for facility fixed effects.
